# Visible light reprograms MSCs and T cells into tumor-suppressive states via OPN4-mediated epigenetic remodeling

**DOI:** 10.1038/s41401-026-01851-4

**Published:** 2026-07-06

**Authors:** Yin-zhi Xu, Zhao-yuan Xu, Uma K. Aryal, Andy Chen, Sungsoo Na, Hiroki Yokota

**Affiliations:** 1 Weldon School of Biomedical Engineering, Purdue University Indianapolis, Indianapolis, IN 46202 USA; 2 Department of Biochemistry and Molecular Biology, Ferguson College of Agriculture, Oklahoma State University, Stillwater, OK 74075 USA; 3 Department of Comparative Pathobiology, College of Veterinary Medicine, Purdue University, West Lafayette, IN 47907 USA; 4 Department of Medical Genetics, Simon Comprehensive Cancer Center, Indiana University School of Medicine, Indianapolis, IN 46202 USA; 5 Department of Anatomy, Cell Biology, and Physiology, Indiana Center for Musculoskeletal Health, Indiana University School of Medicine, Indianapolis, IN 46202 USA

## Abstract

Cellular metabolism critically influences tumor resistance. Biophysical stimulations such as mechanical vibration and electrical pulses can reprogram mesenchymal stem cells (MSCs) and T cells into tumor-suppressive states. Here, we investigated whether optical pulses (OP) could similarly promote an anti-tumor microenvironment. Using mechanical and electrical stimulation as controls, we examined optically stimulated MSCs and T cells and their conditioned medium (CM) in breast cancer and osteoclast models. OP elicited color-specific effects: blue light induced direct tumor cell death, whereas green and red light converted MSCs into induced tumor-suppressing (iTS) cells. This conversion was mediated by OPN4, independent of the Piezo1 pathway engaged by mechanical and electrical cues. Green/red pulses enhanced nucleosome scattering, increased the NAD⁺/NADH ratio, reduced the repressive histone mark H3K9me3, and activated demethylases KDM3A/KDM4. Proteomic profiling revealed enrichment of transferrin receptor (TFRC) and Annexin A2 (ANXA2) in CM, which may suppress tumors via CD44 interaction and are associated with reduced immune-evasive signaling, including Programmed Death-Ligand 1 (PD-L1). These findings demonstrate that visible light pulses can epigenetically reprogram MSCs and T cells, offering a potential therapeutic strategy for breast cancer and bone metastasis.

## Introduction

Cellular responses to biophysical cues have emerged as a promising strategy for reprogramming immune and stromal cells toward tumor-suppressive phenotypes [1–3]. Mechanical vibration, such as low-intensity vibration (LIV) [4] and electrical stimulation (ES) [5], has been shown to generate induced tumor-suppressing (iTS) cells from mesenchymal stem cells (MSCs) and T cells. In contrast, the potential of optical stimulation to elicit similar effects remains largely underexplored. iTS cells secrete tumor-suppressive factors [6] and can be generated through diverse methods, including oncogene overexpression and activation of proliferative signaling pathways, yet biophysical stimulation offers the advantage of being noninvasive and avoiding toxic compounds.

Light, as a versatile and non-invasive stimulus, holds unique potential for wavelength-specific modulation of cellular states [7–9]. In cancer therapy, light has been employed to activate light-sensitive materials that release therapeutic agents upon irradiation [10, 11], or to generate localized heat capable of ablating tumor cells [12, 13]. Building on this foundation, we investigated whether visible light pulses, specifically in the blue, green, and red spectra, could reprogram MSCs and T cells into iTS cells, and thereby reconfigure the tumor and bone microenvironments.

In this study, we investigated whether visible light pulse irradiation can induce the generation of iTS cells. Given the close relationship between cellular metabolism, chromatin dynamics, and immune regulation, we further examined whether optical stimulation alters metabolic state and immune-related signaling during iTS cell generation. If so, what irradiation parameters most significantly influence the efficiency of this conversion? To address this, we systematically evaluated key variables including wavelength (color), pulse frequency, brightness (measured in lux), and irradiation duration. Previous studies have shown that LIV and ES reprogram MSCs and T cells into iTS cells via the activation of the mechanosensitive ion channel Piezo1 [5]. Here, we explored whether Piezo1 also mediates the effects of optical stimulation, or whether alternative receptors, such as the light-sensitive opsin, OPN4, play a role in this process. LIV and ES were used as positive controls for Piezo1-mediated reprogramming. Furthermore, global proteomics analysis of CM identified novel tumor-suppressing proteins, which were uniquely enriched in CM derived from optically stimulated MSC-derived iTS cells.

To further understand the role of epigenetic regulation in iTS cells, we investigated whether the generation of iTS cells is accompanied by alterations in chromatin architecture, with a particular focus on nucleosome clustering. Given that iTS cells exhibit elevated metabolic activity and that chromatin organization is intimately connected to cellular plasticity and metabolic state [14–16], we hypothesized that light-induced reprogramming may entail epigenetic remodeling. Specifically, we examined the histone modification H3K9me3, whose upregulation is associated with transcriptional repression [17, 18]. Because iTS cell generation involves the activation of proliferative signaling pathways, we further hypothesized that chromatin deconditioning, characterized by nucleosome scattering, may play a key role in this cellular transition.

In this study, we examined the functional impact of optically-generated iTS cells and their CM on two critical cellular targets within the tumor–bone microenvironment: breast cancer cells and bone-resorptive osteoclasts. These cell types were selected due to their central roles in driving tumor progression and skeletal degradation, particularly in the context of bone-metastatic breast cancer [19, 20]. By assessing changes in cancer cell viability, proliferation, and migration, as well as osteoclast differentiation and resorptive activity, we aimed to determine the extent to which optically reprogrammed iTS cells exert tumor-suppressive and bone-protective effects. Our findings provide compelling evidence that visible light stimulation, when applied under optimized conditions, can generate iTS cells capable of modulating both tumor and stromal components of the microenvironment. These results highlight the potential of color-specific optical stimulation as a precision biophysical strategy for eliciting targeted anti-tumor responses while simultaneously preserving bone integrity. This approach offers a non-invasive, tunable platform for therapeutic intervention, with broad implications for the treatment of cancers that metastasize to the bone.

## Materials and methods

### Cell lines and reagents

EO771 mouse mammary tumor cells (CH3 BioSystems, Amherst, NY, USA), 4T1.2 mouse mammary tumor cells (obtained from Dr. R. Anderson at Peter MacCallum Cancer Institute, Melbourne, Australia), and MDA-MB-231 and MDA-MB-436 breast cancer cells (ATCC, Manassas, VA, USA) were cultured in DMEM (Gibco, Carlsbad, CA, USA). RAW264.7 pre-osteoclast cells (ATCC, Manassas, VA, USA) were grown in αMEM (Gibco, Carlsbad, CA, USA), while Jurkat and EL4 T cells (ATCC, Manassas, VA, USA) were cultured in RPMI-1640 (Gibco, Carlsbad, CA, USA). Murine mesenchymal stem cells (MSCs; passages 2–8), derived from the bone marrow of the C57BL/6 strain, were cultured in MesenCult culture medium (Stem Cell Technology, Cambridge, MA, USA). The culture medium was supplemented with 10% fetal bovine serum and antibiotics (50 units/mL penicillin and 50 μg/mL streptomycin), and cells were maintained at 37 °C and 5% CO_2_. Paclitaxel (Taxol, #3257, Tocris Bioscience, Minneapolis, MN, USA) was applied to breast cancer cells at 0.01–100 μM, and cells were cultured for 2 days before testing cell viability. Chaetocin (Sigma-Aldrich, St. Louis, MO, USA), a histone methyltransferase inhibitor, was used at 10 nM, while Trichostatin A (TSA; Sigma-Aldrich), a histone deacetylase inhibitor, was used at 40 nM. In addition, we also employed 1–5 µg/mL of recombinant human annexin A2 (ANXA2) and transferrin receptor (TFRC) (ACRO Biosystems, Newark, DE, USA).

### Application of biophysical stimuli

Low-intensity vibration (LIV) was applied to MSCs and T cells using a previously described protocol and a custom-designed vibration apparatus [4]. Mechanical shaking was performed with an MTS 2/4 digital microtiter shaker (IKA Works, NC, USA). To modulate the viscosity of the culture medium, methylcellulose was added as previously reported [21]. The viscosity of the control medium was estimated at 0.8 centipoise (cP). Electrical stimulation (ES) was applied using the previously established method [5]. We utilized a pair of platinum wires (240 µm diameter), which were positioned in parallel with a 2 cm gap and immersed in the culture medium. Optical pulses (OP) were delivered using high-power LEDs emitting at 470 nm (blue), 530 nm (green), and 625 nm (red) wavelengths (LED4D022, Thorlabs, Newton, NJ, USA). Pulse modulation was controlled via an LED driver (DC4104, Thorlabs), coupled with a function generator (AFG-2005, GW Instek, New Taipei City, Taiwan, China). Light intensity was quantified in lux using a digital lux meter (LX1330B, Thoursandshores Inc., Newark, CA, USA). Cells received LIV, ES, or OP for 1 h unless otherwise indicated.

### Preparation of conditioned medium (CM)

Approximately 3 × 10^5^ MSCs or T cells were seeded in a 35-mm tissue culture dish or petri dish, respectively, and cultured overnight to reach 70%–80% confluence. Cells were subjected to one of the biophysical stimulations (LIV, ES, or OP). Following stimulation, cells were washed twice with phosphate-buffered saline (PBS) to remove residual serum components and incubated in serum-free medium. After 48 h, the CM was collected and centrifuged at 2000 rpm for 10 min at 4 °C to remove detached cells. The supernatant was further centrifuged at 3000 × *g* for 10 min to eliminate residual debris. The resultant medium was treated with a 0.22 µm polyethersulfone membrane filter (Sigma-Aldrich). The filtered CM was aliquoted and stored at −80 °C until use. For in vitro functional assays, CM was mixed with fresh complete culture medium at a final concentration of 50% (*v*/*v*), unless otherwise specified. At least three independent CM preparations were used for each experiment to ensure reproducibility.

### MTT assay, CCK8 assay, transwell invasion assay, and scratch assay

Cell viability and proliferation were assessed using the MTT assay and CCK8 assay (Thermo Fisher, Waltham, MA, USA). For the MTT assay, metabolic activity was quantified by measuring absorbance at 570 nm with a multi-well spectrophotometer. For the CCK-8 assay, cell viability was quantified by measuring absorbance at 450 nm with a multi-well spectrophotometer. Cell motility was evaluated using a transwell invasion assay, while two-dimensional migration was assessed via a wound-healing scratch assay.

### Western blot analysis

Cells were lysed in RIPA buffer supplemented with protease and phosphatase inhibitors. Protein concentrations were measured using a BCA assay (Thermo Fisher). Equal amounts of protein were separated on 10%–15% SDS-PAGE gels and transferred to PVDF membranes (MilliporeSigma, Burlington, MA, USA). Membranes were blocked for 1 h, incubated overnight with primary antibodies, and then with HRP-conjugated secondary antibodies (Cell Signaling) for 45 min. Signals were detected using SuperSignal West Femto substrate (Thermo Scientific) and quantified with a LAS-3000 imaging system (Fujifilm, Tokyo, Japan). Antibodies against AKT, p-AKT, ERK, p-ERK, Src, p-Src, Cmyc, KDM4, Ezh2, snail, β-catenin, Anxa2, Tfrc, CD44 (Cell Signaling; Danvers, MA, USA), KDM3A, Piezo1 (Proteintech, Rosemont, IL, USA), RANKL (Invitrogen, Carlsbad, CA, USA), ALP, OPN4 (Santa Cruz, Paso Robles, CA, USA), H3K9me3 (Active Motif, Carlsbad, CA, USA), and β-actin (Sigma-Aldrich) were used. The secondary antibodies were HRP-linked anti-mouse IgG (#7076, Cell Signaling) and anti-rabbit IgG (#7074, Cell Signaling).

### siRNA transfection

For silencing PIEZO1 and OPN4, cells were transfected with target-specific siRNAs (Thermo Fisher) using Lipofectamine™ RNAiMAX transfection reagent (13778100, Thermo Fisher) according to the manufacturer’s protocol. Briefly, siRNAs were diluted in Opti-MEM™ Reduced Serum Medium (31985062, Thermo Fisher) and mixed with Lipofectamine™ RNAiMAX at a final concentration of 50 nM siRNA. After 15–20 min of incubation at room temperature, the complexes were added dropwise to cultured cells in antibiotic-free medium. A non-targeting negative control siRNA (4390843, Thermo Fisher) was used in parallel. Transfected cells were collected 48–72 h post-transfection for subsequent functional assays and Western blot validation.

### 3D spheroid and ex vivo tissue assays

Breast cancer cells were seeded at 1 × 10⁴ cells/well in ultra-low attachment 96-well plates (S-BIO, Constantine, MI, USA) to form spheroids. Images were captured every 24 h, and spheroid cross-sectional areas were quantified using ImageJ. For the ex vivo bone assay, femur samples were cultured in DMEM supplemented with 10% fetal bovine serum and antibiotics for 24 h, followed by treatment with MSC-derived iTS CM for two weeks. Gene expression changes were then analyzed.

### Osteoclast differentiation assay

RAW264.7 pre-osteoclast cells (1 × 10³ cells/well) were seeded in 96-well plates and cultured in α-MEM with 10% FBS and 1% penicillin-streptomycin. Osteoclastogenesis was induced by adding recombinant mouse RANKL (50 ng/mL) and incubating for 5–6 days at 37 °C with 5% CO_2_. To assess the effect of CM, the culture medium was replaced with CM on day 2 and maintained for 4 days, with the medium refreshed every 2 days. Cells were fixed and stained for TRAP. TRAP-positive multinucleated cells (≥3 nuclei) were counted as mature osteoclasts in five random fields per well, in triplicate.

### Proteomics sample preparation for secretome analysis

For secretome analysis, protein concentration in the culture medium was measured using the Pierce BCA (Bicinchoninic Acid) assay kit (Thermo Scientific). The culture volume equivalent to containing 20 µg of total protein was separated and precipitated using four volumes of cold (−20 °C) acetone, the mixture was vortexed and then incubated overnight at −20 °C. After incubation, precipitated protein pellets were collected by centrifugation at 20,000 × *g* at 4 °C for 15 min. The supernatant was removed, and the protein pellet was washed twice with 80% cold (−20 °C) acetone, and the clean and washed protein pellets were briefly dried in the CentriVap for 1–2 min (Thermo Fisher). The dried pellets were re-suspended in 40 µL of 8 M urea and incubated at room temperature with continuous vortexing for 60 min for complete solubilization of protein pellets. After 60 min, DTT (dithiothreitol) was added to each sample at a final concentration of 10 mM (1.02 µL of 500 mM DTT). The samples were incubated at 37 °C for 45 min for reducing disulfide bonds, and then cysteine alkylated by treating with iodoacetamide (IAA) to a final concentration of 20 mM (2.13 µL of 500 mM IAA stock). The alkylation was allowed to proceed by incubating samples in the dark at room temperature for 45 min. Following this, trypsin was added at a 1:20 enzyme-to-protein ratio (1 µg of enzyme per 20 µg protein), the solution volume was adjusted to 100 µL using 50 mM of ABC. After adding 3 µL of acetonitrile (ACN), the samples were incubated overnight at 37 °C with shaking in a Thermomixer for digestion. Finally, 1.0 µL of trifluoroacetic acid (TFA) was added to the samples to stop digestion. The samples were centrifuged at 20,000 × *g* for 15 min at 4 °C to remove any remaining/undigested debris, and digested peptides were desalted using Pierce C18 spin columns (Thermo Fisher) using manufacturer’s protocol as described previously [22].

### LC-MS/MS analysis

Clean and dried peptides were dissolved in 3% acetonitrile and 0.1% FA and loaded onto Evotips Pure (EvoSep) according to the manufacturer’s instructions. Peptides were then analyzed using the Evosep One LC system (EvoSep) coupled with a timsTOF HT mass spectrometer (Bruker). The 60 SPD (sample per day) method was used with the PepSep C18 column (8 cm × 150 μm, 1.5 μm; Bruker). The columns were connected to a ZDV (zero dead volume) Sprayer 20 μm emitter (Bruker) inside a Captive Spray source (Bruker). The mobile phases included 0.1% FA in LC-MS grade water as solvent A and 0.1% FA in LC–MS grade acetonitrile as solvent B. The TIMS (trapped ion mobility mass spectrometry) dimension was calibrated using three Agilent ESI tuning mix ions (622.0289, 0.9848 V/cm^2^; 922.0097, 1.1895 V/cm^2^; 1221.9906, 1.3820 V/cm^2^) in positive mode. For the data dependent acquisition (DDA), DDA-PASEF (DDA-parallel accumulation serial fragmentation) mode was used to perform data acquisition of the samples on the timsTOF HT. Ten PASEF/MS/MS scans were acquired per cycle using the 60 SPD method. Spectra were acquired within an *m*/*z* range from 100 to 1700 and ion mobility (IM) from 0.6 to 1.6 V/cm^2^. The ion mobility-dependent collision energy was set from 59 eV at 1.6 V/cm^2^ to 20 eV at 0.6 V/cm^2^. Singly charged precursors were excluded by their position in the *m*/*z*-IM plane, and a precursor signal intensity threshold of 2500 was used for fragmentation. Precursors were isolated with a 2 Th window (<700 Da) and a 3Th window (>800 Da) and were excluded for 0.4 min when they were over a target intensity of 20,000.

For data-independent acquisition (DIA), dia-PASEF mode was used. The isolation windows covered an *m*/*z* range from 400 to 1200, and the IM was set from 0.6 to 1.6 V/cm^2^. The dia-PASEF method contained 16 dia-PASEF scans with a cycle time of 1.8 s. The ramp time was set as 100 ms, and the collision energy was set from 59 eV at 1.6 V/cm^2^ to 20 eV at 0.6 V/cm^2^.

### Proteomics data analysis

LC-MS/MS raw data were processed with MaxQuant (v1.6.3.3) against the Uniprot mouse protein database, allowing up to two missed cleavages and applying a 1% false discovery rate. Relative protein quantification was based on MS1 intensity-based label-free quantitation, and only proteins with at least one unique peptide and two MS/MS counts were included in the analysis. Proteins identified with non-zero LFQ (label-free quantitation) values in at least two of the three biological replicates in one treatment group were considered for statistical analysis (see below). To assess tumor-suppressive potential, we tested recombinant proteins, including TFRC and ANXA2, by adding 1–5 μg/mL of each to tumor cells in an MTT assay and measuring cell viability.

### Stochastic Optical Reconstruction Microscopy (STORM)

We use MSCs transfected with pBREBAC-H2B-Halo plasmid (#91564; Addgene; Watertown, MA, USA) by Lipofectamine3000 (L3000008; Thermo Fisher) for STORM imaging. JF646 (GA1120; Promega, Madison, WI, USA) was used to bind with Halo-tagged H2B protein, and will be excited under 640 nm pulses. We use an Olympus IX83 microscope (IX3-LBD; Olympus, Hachioji, Japan) with a 20 mW 640 nm laser (Excelitas Technologies, Wiesbaden, Germany) and a 60 × 1.25 NA oil-immersion objective lens for imaging. Images were captured by an ORCA-Fusion sCMOS camera (C14440-20UP; Hamamatsu Photonics, Hamamatsu, Japan). We captured 2000 frames for each nucleus (100 frames per second). High-resolution localization of individual fluorescent molecules was achieved using a point spread function (PSF), thereby enabling quantitative analysis of chromatin architecture. The degree of nucleosome clustering was assessed by spatial statistics based on Besag’s L-function, expressed as “*L*(*r*) - *r*”. This metric reflects the number of nucleosomes within a given radius (*r*), where higher values indicate a more compact chromatin state.

### Immunofluorescence (IF) staining

Cells were fixed with 4% paraformaldehyde, permeabilized with 0.1% Triton X-100, and blocked in 5% BSA. Primary antibodies were applied overnight at 4 °C, followed by incubation with fluorophore-conjugated secondary antibodies. Samples were mounted in Fluoromount-G containing DAPI for microscopy.

### Fluorescence Resonance Energy Transfer (FRET) imaging

To visualize histone modifications in live cells, an H3K9me3 biosensor [23] (120802, Addgene) was transfected in MDA-MB-231 cells using Lipofectamine 2000 (Invitrogen) following the manufacturer’s protocol. The transfected cells were incubated at 37 °C and 5% CO_2_ for 36–48 h prior to imaging. FRET imaging was performed using a Nikon Ti-E inverted microscope equipped with an EMCCD camera (Photometrics, Tucson, AZ, USA), a Perfect Focus System (Nikon, Tokyo, Japan) that maintains focus in real time (Nikon), a filter wheel controller, and the following filter sets: a CFP excitation filter at 438/24 nm; a CFP emission filter at 483/32 nm; and a FRET emission filter at 542/27 nm. Images were acquired with a 40´ objective (0.75 numerical aperture) (Nikon). The pixelwise ratio images of FRET emission to CFP emission were captured and generated by NIS-Elements software (Nikon) to visualize H3K9me3 activity.

### Co-immunoprecipitation (co-IP)

Cells were lysed on ice in NP-40 buffer (50 mM Tris-HCl, pH 7.4, 150 mM NaCl, 1% NP-40, 1 mM EDTA) supplemented with protease/phosphatase inhibitors for 30 min and centrifuged (12,000 × *g*, 10 min, 4 °C). Recombinant human TFRC or ANXA2 proteins (rTFRC, rANXA2; ACRO Biosystems) were added to the lysates and pre-incubated at 4 °C for 30 min. An equal amount of proteins (0.5–1.0 mg per IP) was pre-cleared with Protein A/G magnetic beads (20 µL, 30 min, 4 °C) and incubated with anti-CD44 primary antibody (1–2 µg, Cell Signaling) or isotype IgG (negative control) overnight at 4 °C with rotation. Immune complexes were captured with Protein A/G beads (20–30 µL, 1–2 h, 4 °C), washed 4–5 times with cold lysis buffer, containing 300 mM NaCl for the first wash and 150 mM thereafter, and eluted by boiling in 2× SDS sample buffer (95 °C, 5 min). Inputs (5%–10% of lysate) and IP eluates were analyzed by SDS-PAGE and Western blot analysis using antibodies against TFRC and ANXA2.

### Statistical analysis

For cell-based experiments, data are presented as mean ± standard deviation (SD) from at least three independent biological replicates. Normality of datasets was assessed using the Shapiro–Wilk test prior to statistical comparisons. Statistical significance was determined using an unpaired, two-tailed Student’s *t*-test. In figure panels, single, double, and triple asterisks denote *P* < 0.05, *P* < 0.01, and *P* < 0.001, respectively.

## Results

### Optical pulses (OP) stimulation reprogramed MSCs into tumor-suppressive iTS cells

Our previous work demonstrated that mechanical and electrical cues can reprogram MSCs into iTS cells. Specifically, low-intensity vibration (LIV) and electrical stimulation (ES) generated MSC-derived secretomes that suppressed breast cancer cell viability, motility, invasion, and spheroid growth, highlighting the feasibility of using physical stimulation to induce tumor-suppressive functions (Supplementary Fig. S1–S4).

**Fig. 1:**
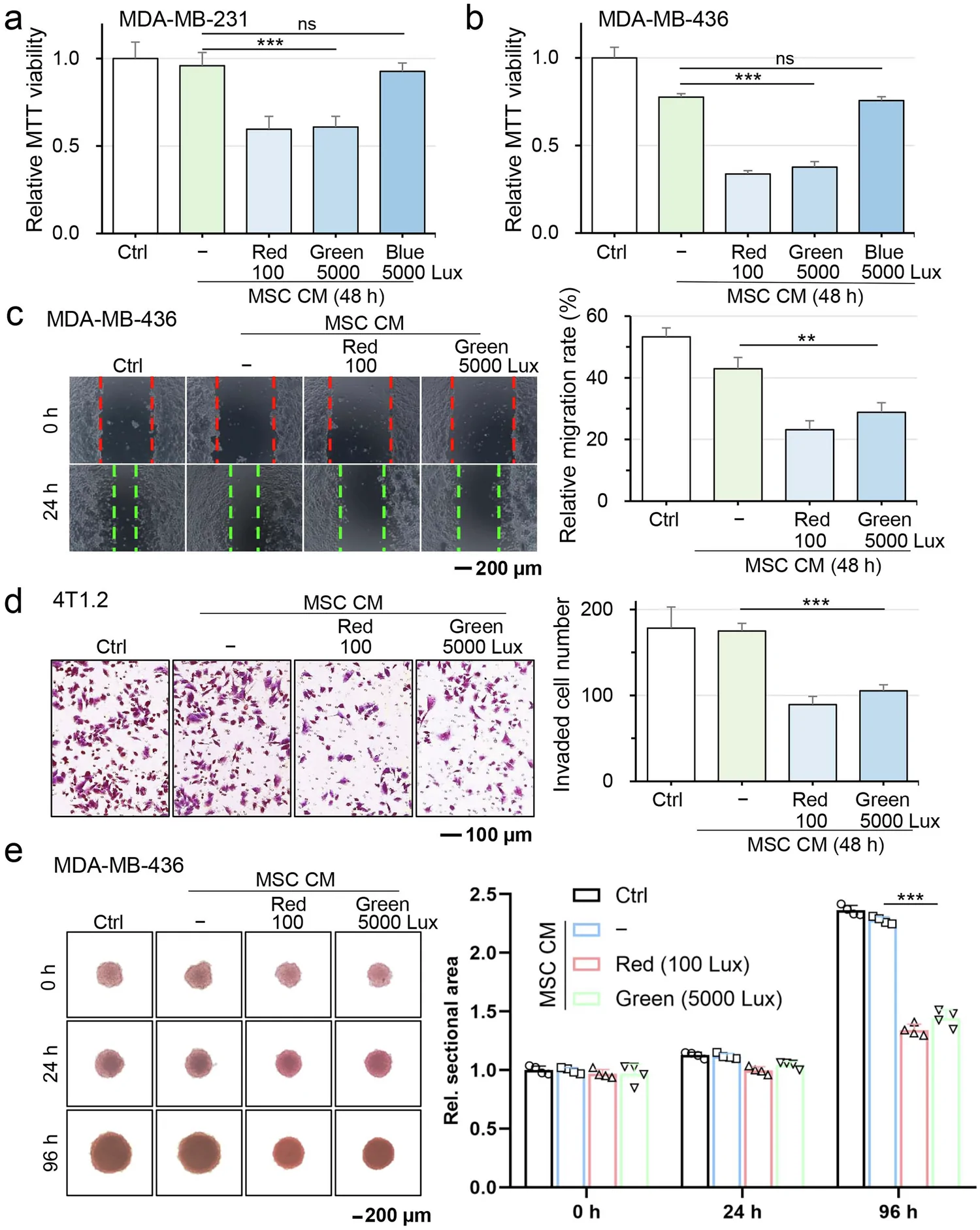
Optical pulse stimulation reprograms MSCs into induced tumor-suppressive (iTS) cells. MSCs were exposed to pulsed red (100 Lux), green (5000 Lux), or blue (5000 Lux) light at 10 Hz for 1 h. Conditioned medium (CM) was collected after 48 h. **a**, **b** CM from MSCs stimulated with green or red light significantly reduced MTT-based viability of MDA-MB-231 and MDA-MB-436 breast cancer cells, respectively, after 48 h. Blue light-treated CM had no detectable effect. **c**, **d** Green or red light-treated MSC-derived CM inhibited the 2D motility of MDA-MB-436 cells and suppressed the transwell invasion of MDA-MB-231 cells within 24 h. **e** CM from red and green light-stimulated MSCs significantly reduced the size of MDA-MB-436 tumor spheroids over 96 h. **P < 0.01, ***P < 0.001, ns not statistically significant. Ctrl control; and CM conditioned medium.

These findings prompted us to further optimize stimulation parameters and explore additional modalities with higher precision and controllability. To this end, we tested whether OP stimulation could similarly convert MSCs into tumor-suppressive iTS cells. MSCs were exposed to red (100 Lux), green (5000 Lux), or blue (5000 Lux) light pulses at 10 Hz for 1 h, followed by incubation for 48 h before CM collection. CM derived from red- or green-light–stimulated MSCs significantly decreased the viability of both MDA-MB-231 and MDA-MB-436 breast cancer cells compared to control CM, whereas blue-light–derived CM showed no significant effect (Fig. 1a, b). In addition, red- and green-light–derived CM markedly inhibited the 2D motility of MDA-MB-436 cells within 24 h (Fig. 1c). Consistently, transwell invasion assays demonstrated a significant reduction in 4T1.2 cell invasiveness upon treatment with CM from red- or green-light–stimulated MSCs (Fig. 1d). Furthermore, tumor spheroid formation of MDA-MB-436 cells was substantially suppressed by CM collected from MSCs treated with red or green light pulses (Fig. 1e). Besides MSCs, T cells were also converted into iTS cells in an OP color-dependent manner (Supplementary Figs. S5 and S6). Collectively, these results indicate that OP stimulation, particularly with red and green light, effectively reprograms MSCs and T cells into iTS cells.

### Mechanical and electrical stimulation induced tumor-suppressive reprogramming of T cells

**Fig. 2:**
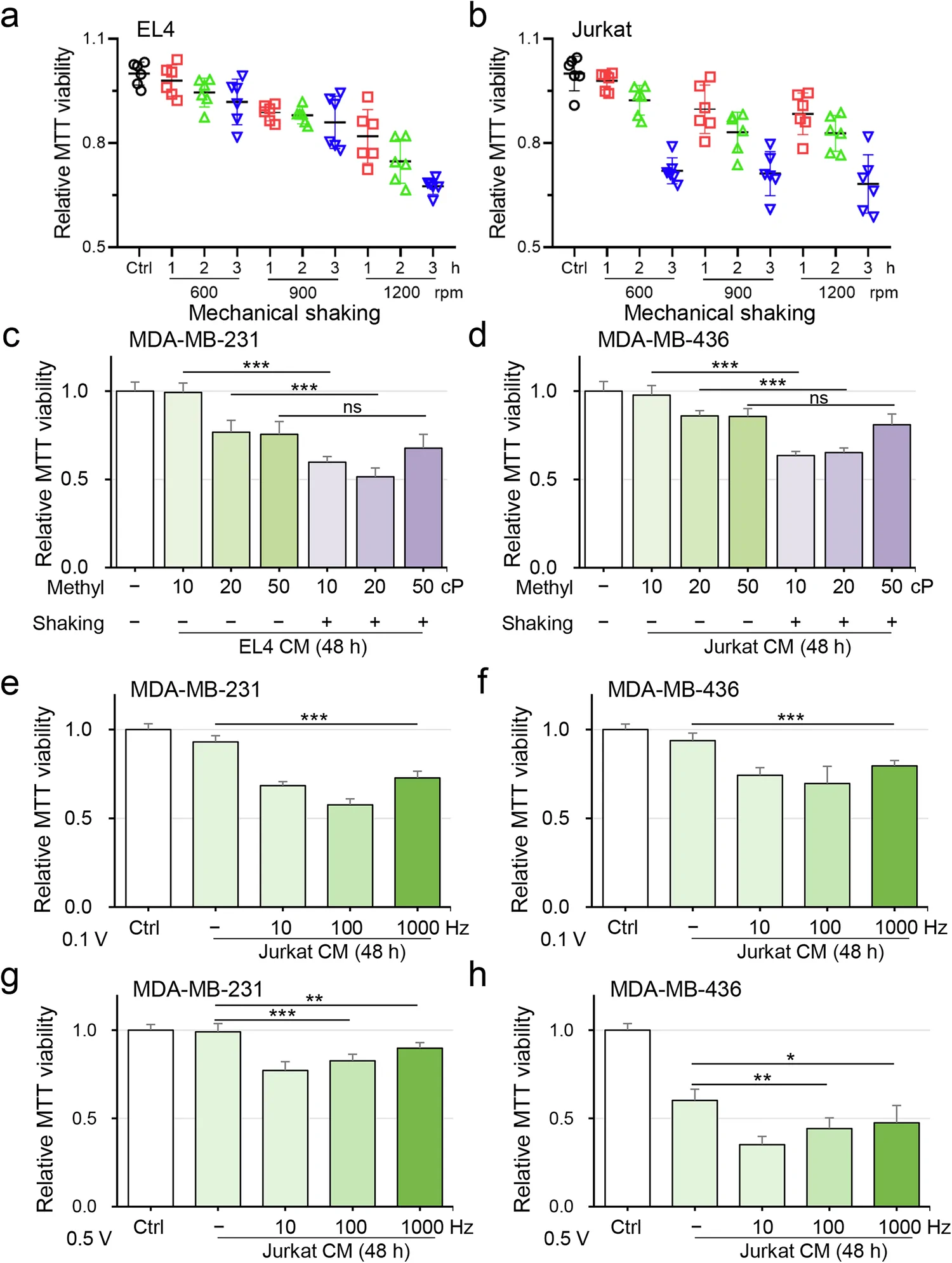
Mechanical and electrical stimulation reprograms T cells into induced tumor-suppressive (iTS) cells. Human Jurkat T lymphocytes and mouse EL4 T lymphocytes were subjected to mechanical shaking or electrical stimulation (ES). Mechanical shaking of EL4 (**a**) or Jurkat cells (**b**) at 600, 900, or 1200 rpm for 1–3 h reduced MTT-based viability in a speed- and time-dependent manner. CM from mechanically shaken EL4 (**c**) and Jurkat cells (**d**), with methylcellulose-based viscosity modulation, significantly reduced MTT-based viability of MDA-MB-231 or MDA-MB-436 cells in a viscosity-dependent manner. CM from ES-treated Jurkat cells (0.1 V) significantly reduced the viability of MDA-MB-231 (**e**) and MDA-MB-436 (**f**) breast cancer cells after 48 h. (**g** and **h**) CM from Jurkat cells stimulated with ES at 0.5 V enhanced tumor-suppressive effects on MDA-MB-231 and MDA-MB-436 cells. *P < 0.05, **P < 0.01, ***P < 0.001, ns not statistically significant. Ctrl control, and CM conditioned medium.

To explore whether mechanical and electrical stimulation can also reprogram T cells into tumor-suppressive effector cells, we first exposed EL4 and Jurkat T cells to orbital shaking at 600, 900, or 1200 rpm for durations of 1–3 h. MTT assays revealed a stimulation intensity- and time-dependent reduction in cell viability, suggesting increased mechanical stress compromises T cell metabolic activity (Fig. 2a, b). Next, we tested whether methylcellulose-assisted mechanical stimulation could induce tumor-suppressive secretory functions. EL4 or Jurkat cells were cultured in 0, 10, 20, or 50 cp methylcellulose and subjected to shaking or left unstimulated. CM collected 48 h later was applied to MDA-MB-231 or MDA-MB-436 breast cancer cells. MTT assays showed that CM from 10 and 20 cp methyl-shaken cells moderately suppressed cancer cell viability (Fig. 2c, d). We next tested whether ES could enhance the tumor-suppressive capacity of T cells. Jurkat cells were stimulated at 0.1 V with 10, 100, or 1000 Hz. CM derived from 100 Hz stimulation showed the strongest inhibitory effect on both MDA-MB-231 and MDA-MB-436 cell viability, followed by 10 Hz, and with minimal effect at 1000 Hz (Fig. 2e, f). When the stimulation voltage was increased to 0.5 V, the most effective frequency shifted to 10 Hz, with both 100 and 1000 Hz showing weaker inhibition (Fig. 2g, h). These findings indicate that both mechanical and electrical stimuli can reprogram T cells into a tumor-suppressive state in a context- and parameter-dependent manner.

### Red and green pulses induced reprogramming of T cells

**Fig. 3:**
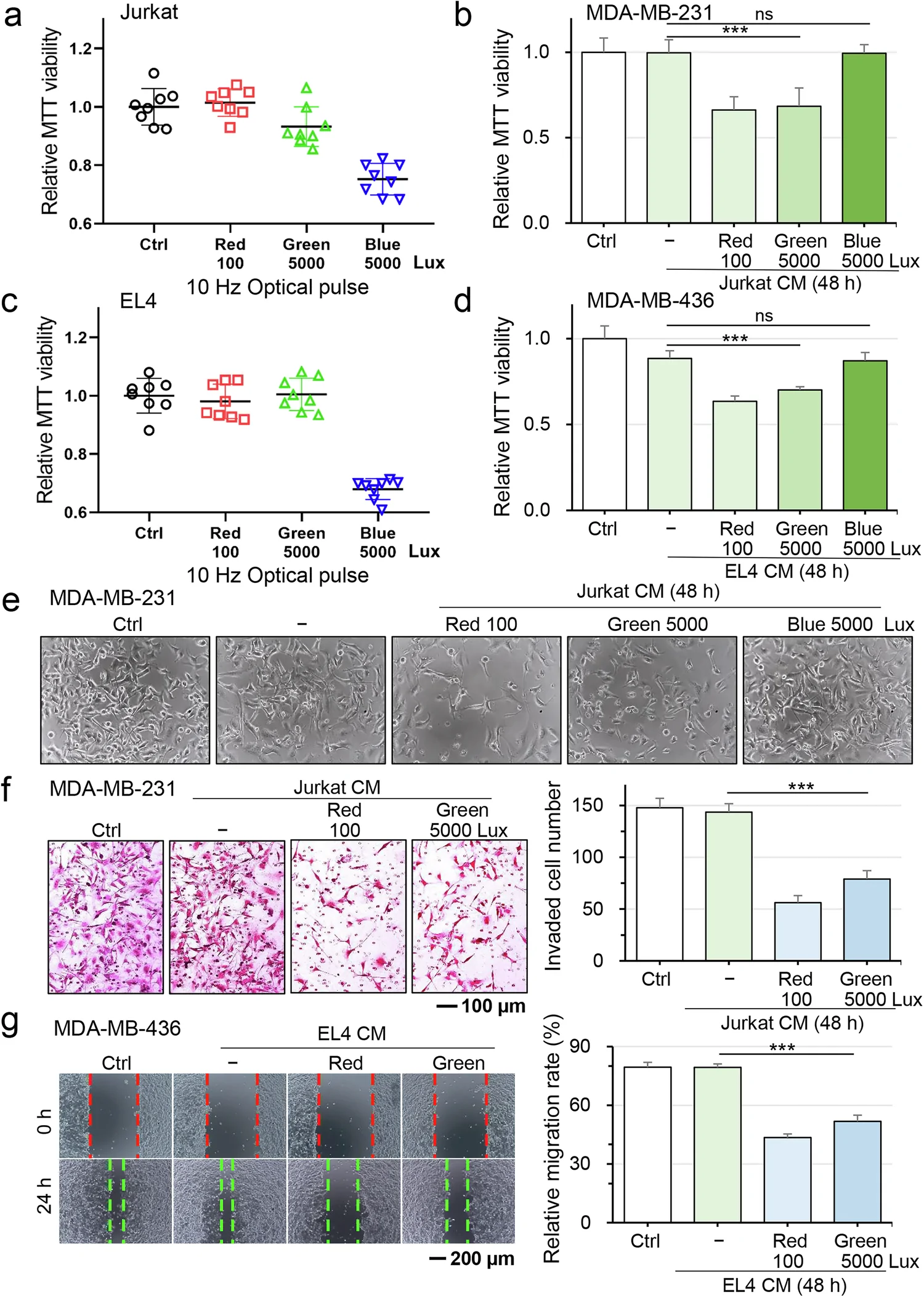
Optical pulses reprogram T cells into iTS cells in a light-spectrum–dependent manner. Jurkat and EL4 T cells were exposed to red (100 Lux), green (5000 Lux), or blue (5000 Lux) pulses at 10 Hz for 1 h, followed by 48 h incubation. MTT assays of Jurkat (**a**) and EL4 cells (**c**) showing minimal cytotoxicity from red and green light, but a significant reduction under blue light. MTT assays of MDA-MB-231 (**b**) or MDA-MB-436 cells (**d**) in response to CM from Jurkat or EL4 cells, showing growth suppression by red- and green-light CM but no effect from blue-light CM. Red- and green-light CM impaired cancer cell viability, invasion, and migration, as shown by phase-contrast images of MDA-MB-231 cells (**e**), Transwell assays of MDA-MB-231 cells (**f**), and scratch wound assays of MDA-MB-436 cells (**g**); blue-light CM had no effect. ***P < 0.001, ns not significant. Ctrl control, CM conditioned medium.

To determine whether optical stimulation can convert T cells into iTS cells, we subjected Jurkat and EL4, to red, green, or blue pulsed at 10 Hz for 1 h. MTT assays showed that red and green light had little cytotoxic effect, while blue light caused a significant reduction in cell viability (Fig. 3a, c and Supplementary Fig. S5). We then collected the CM 48 h after light exposure and assessed its effect on breast cancer cell viability. CM derived from red- and green-light–stimulated Jurkat or EL4 T cells significantly inhibited the growth of MDA-MB-231 or MDA-MB-436 cells (Fig. 3b, d). In contrast, CM from blue-light–stimulated T cells exhibited no anti-tumor effects. Phase-contrast images of MDA-MB-231 cells treated with CM from Jurkat T cells showed that red- and green-light–derived CM markedly reduced cell viability, characterized by abnormal morphology and decreased cell numbers (Fig. 3e), whereas blue-light CM had no effect. Furthermore, red- and green-light–derived CM significantly inhibited cancer cell motility, as evidenced by reduced invasion of MDA-MB-231 cells in the Transwell assay (Fig. 3f) and impaired migration of MDA-MB-436 cells in the scratch wound assay (Fig. 3g). These findings suggest that red and green optical stimulation can reprogram T cells into tumor-suppressive secretory cells, while blue light is cytotoxic and ineffective for this purpose.

### Piezo1 was required for mechanical and electrical stimulation–induced reprogramming of MSCs

**Fig. 4:**
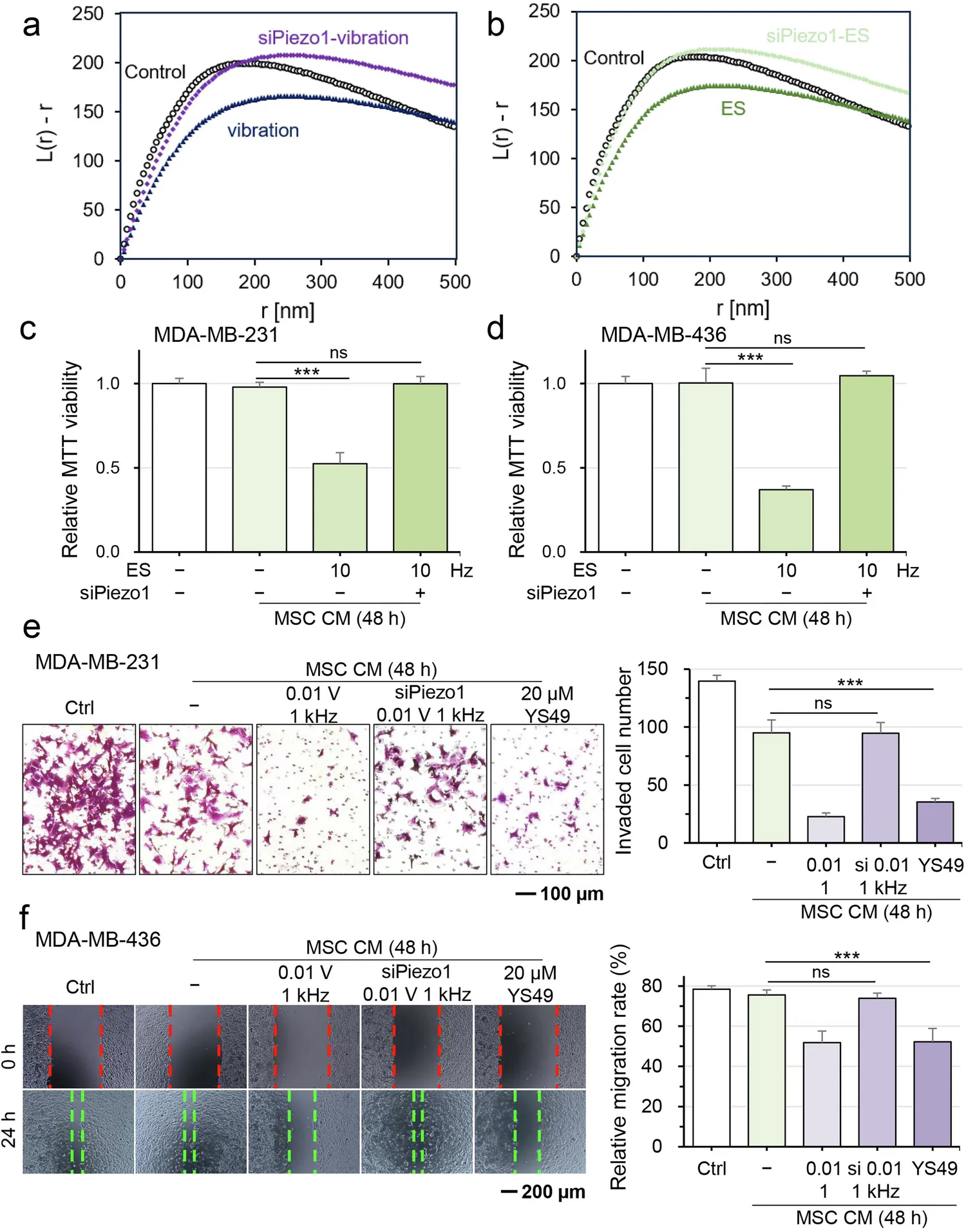
Piezo1 mediates mechanical and electrical stimulation–induced reprogramming of MSCs into tumor-suppressive iTS cells. **a**, **b** Reduction in nucleosome clustering by vibration (LIV) and ES, respectively, with and without Piezo1 knockdown. **c**, **d** Alterations in MTT-based viability in MDA-MB-231 and MDA-MB-436 cells, respectively, in response to MSC CM with and without Piezo1 silencing. **e** Transwell invasion assays of MDA-MB-231 cells treated with ES-stimulated MSC CM. The activation of the PI3K/AKT pathway using YS49 can also enhance the tumor-suppressive capacity of MSC-derived CM. **f** Scratch wound healing assays using MDA-MB-436 cells confirmed that migration inhibition by ES-treated MSC CM was dependent on Piezo1 expression in MSCs. ***P < 0.001, ns not statistically significant. Ctrl control, and CM conditioned medium.

To investigate the molecular mechanism by which LIV and ES reprogram MSCs into iTS cells, we focused on Piezo1, a mechanosensitive ion channel. STORM imaging showed that both vibration and ES (0.1 V, 10 Hz) significantly reduced the value of “*L*(*r*) - *r*”, demonstrating a decrease in nucleosome clustering and indicating the activation of gene expression (Fig. 4a, b). However, Piezo1 knockdown abolished this effect, suggesting that Piezo1 is essential for mechanically induced chromatin remodeling. We next examined whether Piezo1 is necessary for the acquisition of anti-tumor function. MSCs with Piezo1 knockdown were subjected to ES, and CM was collected. CM from ES-treated control MSCs significantly reduced the viability of MDA-MB-231 and MDA-MB-436 cells, while CM from Piezo1-deficient MSCs had no inhibitory effect (Fig. 4c, d and Supplementary Fig. S8a, S8b). In Transwell invasion assays, only CM from control MSCs suppressed MDA-MB-231 invasion after ES. CM from Piezo1-knockdown MSCs did not show this effect (Fig. 4e). Scratch assays confirmed that CM from control but not Piezo1-deficient MSCs inhibited the migration of MDA-MB-436 and 4T1.2 cells (Fig. 4f and Supplementary Fig. S8c). The YS49-driven activation of the PI3K/AKT pathway can also induce a tumor-suppressive phenotype in MSCs, suggesting that multiple signaling pathways contribute to the iTS cells reprogramming process.

### OPN4 was required for light–induced chromatin remodeling and tumor-suppressive function in MSCs and T cells

**Fig. 5:**
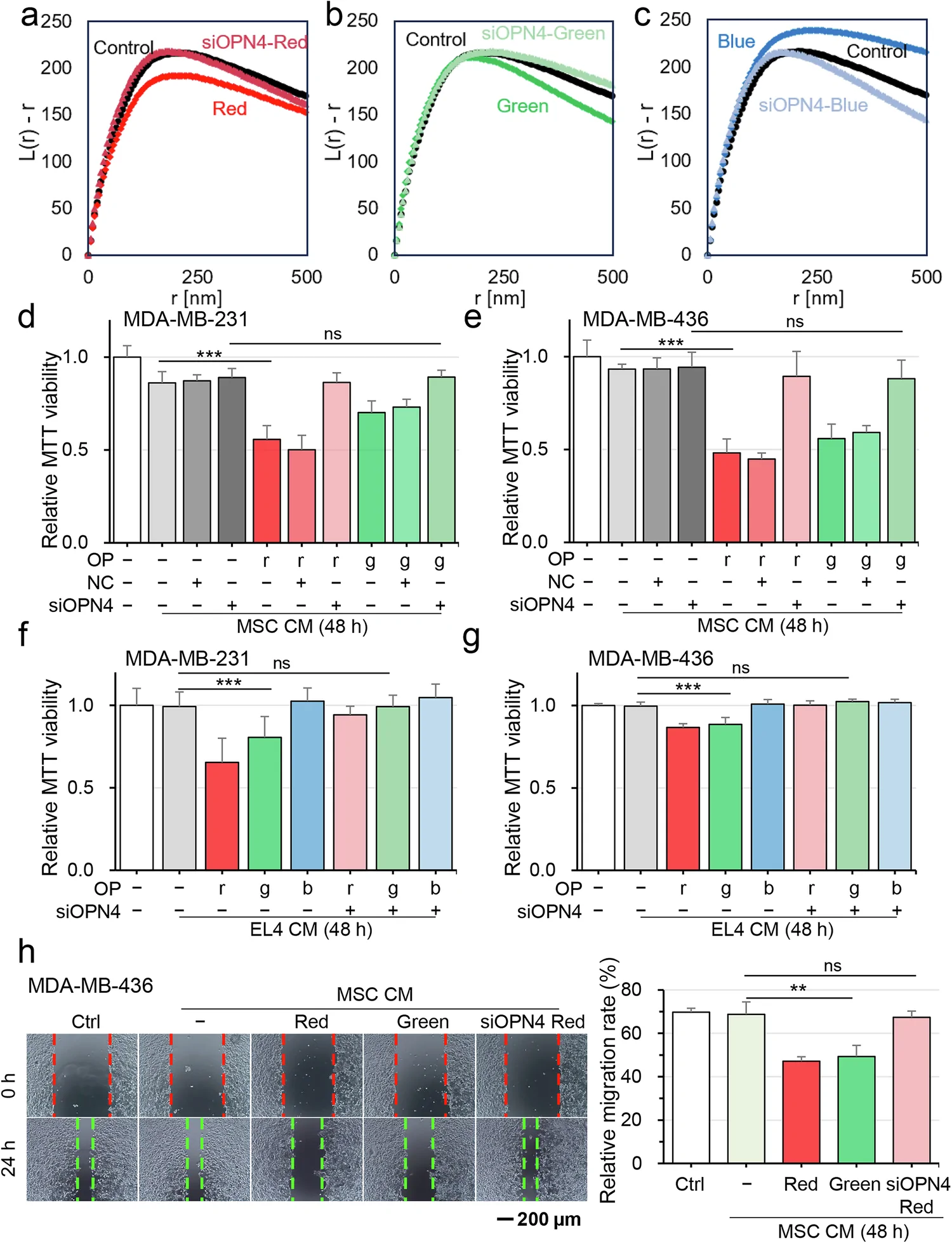
OPN4 mediates red and green light–induced tumor-suppressive reprogramming of MSCs and T cells. **a**–**c** Alteration in nucleosome clustering in response to MSC-derived CM, after red, green, or blue light stimulation (10 Hz, 1 h), respectively, with and without OPN4 knockdown. **d**, **e** MTT-based cell viability of MDA-MB-231 and MDA-MB-436 cells, respectively, in response to red and green pulse-treated MSC-derived CM, with and without OPN4 silencing. **f**, **g** MTT-based cell viability of MDA-MB-231 and MDA-MB-436 cells, respectively, in response to red and green pulse-treated EL4 T cell-derived CM, with and without OPN4 silencing. **h** Scratch wound assay of MDA-MB-436 cells treated with CM derived from MSCs subjected to red or green light stimulation, with or without OPN4 knockdown. **P < 0.01, ***P < 0.001, ns not statistically significant. Ctrl control, and CM conditioned medium.

To investigate how MSCs and T cells sense optical cues, we examined OPN4, a light-sensitive G-protein–coupled receptor. STORM imaging showed that red and green light reduced “*L*(*r*) - *r*” values, indicating decreased nucleosome clustering in MSCs, while blue light increased clustering (Fig. 5a–c). OPN4 knockdown abolished these chromatin changes, implicating OPN4 in light-induced epigenetic remodeling. To assess functional relevance, MSCs were grouped as untreated, siNC-transfected, or siOPN4-transfected, and exposed to no light, red light, or green light, generating nine CM types. These were applied to MDA-MB-231 and MDA-MB-436 cells. Without light, CM from siNC MSCs had minimal effect, while CM from siOPN4 MSCs slightly increased tumor viability (Fig. 5d, e). Under red or green light, CM from untreated and siNC MSCs suppressed tumor viability, but CM from siOPN4 MSCs lost this effect, confirming OPN4’s role in mediating light-induced anti-tumor activity. Similarly, OPN4 knockdown in EL4 and Jurkat T cells abolished the anti-tumor effect of CM following red or green light stimulation (Fig. 5f, g and Supplementary Fig. S8d). Scratch wound assays showed that only CM from light-stimulated MSCs with intact OPN4 suppressed MDA-MB-436 migration, while CM from siOPN4 MSCs did not (Fig. 5h).

Interestingly, Piezo1 knockdown blocked the response of MSCs to ES but did not affect their anti-tumor response to red or green light, indicating that Piezo1 is specifically required for the generation of iTS cells in response to ES but not to light pulses (Supplementary Fig. S8e). These findings establish OPN4 as a key sensor for red and green light, essential for reprogramming MSCs and T cells into tumor-suppressive states. Since the mediators involved in generating iTS cells differ between LIV/ES and OP, we investigated whether their combined application could enhance the tumor-suppressive properties of CM derived from iTS cells. The results for the limited combinations suggest a potential additive effect (Supplementary Fig. S7). However, further systematic analyses are required to identify the most effective combinations.

To further investigate the downstream mechanism of OPN4-mediated reprogramming, we examined OP-induced metabolic changes in MSCs. Measurement of the intracellular NAD⁺/NADH ratio revealed that red light significantly increased the ratio compared with untreated control cells, while green light also induced a moderate increase. In contrast, blue light reduced the ratio, indicating a distinct metabolic response. Importantly, OPN4 knockdown abolished the red light-induced increase, demonstrating that this metabolic remodeling depends on OPN4 signaling (Fig. 10a and Supplementary Fig. S10b).

To further assess metabolic changes associated with mitochondrial activity, JC-1 fluorescence staining, which served as an indicator of mitochondrial membrane potential, was performed. Red light stimulation significantly increased mitochondrial membrane potential in MSCs, as indicated by enhanced JC-1 aggregation. This effect was attenuated in OPN4-silenced cells, suggesting that OPN4 is required for light-induced mitochondrial activation (Supplementary Fig. S10c).

### Regulation of H3K9 trimethylation was involved in the generation of iTS cells

**Fig. 6:**
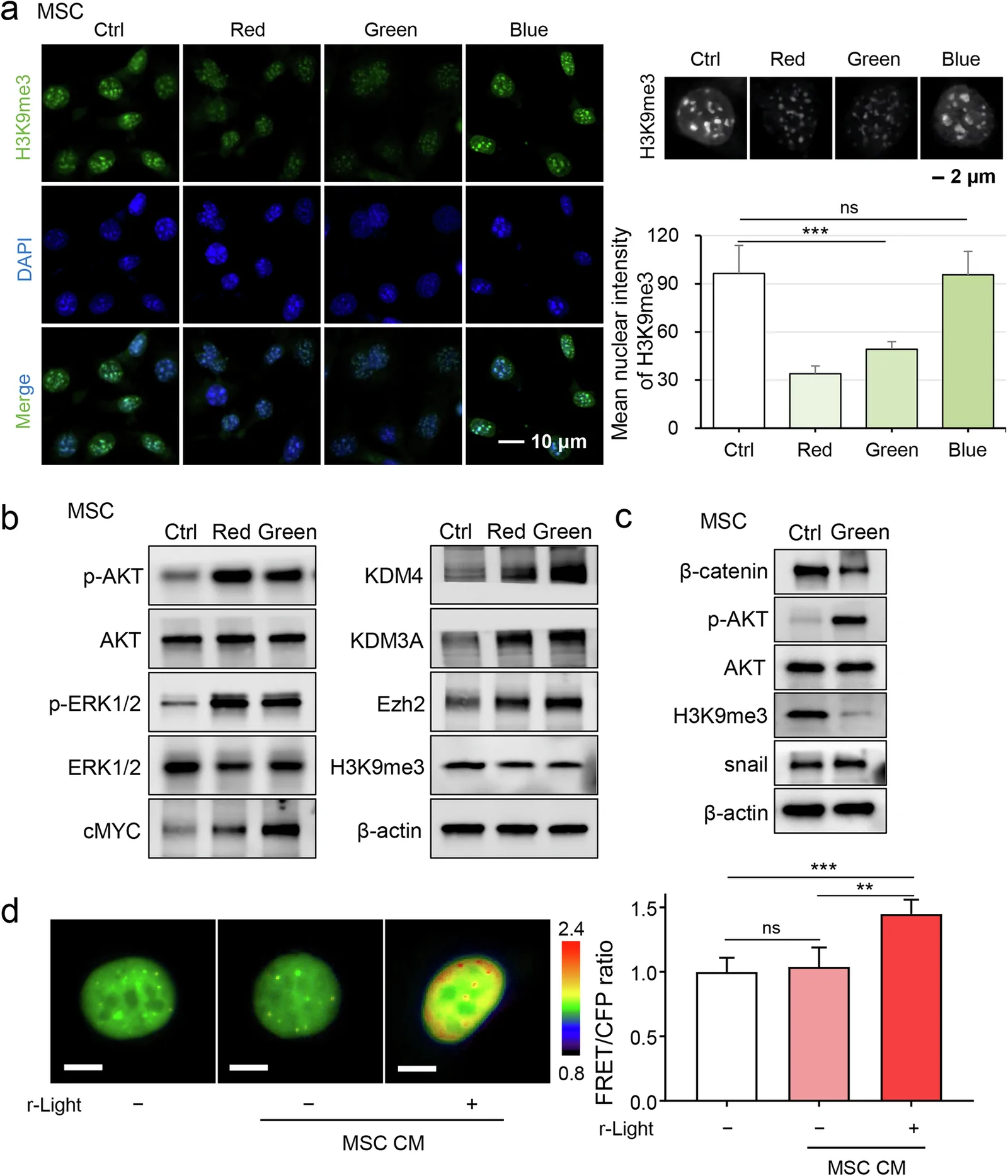
Involvement of H3K9 trimethylation in iTS cell generation. **a** Immunofluorescence of H3K9me3 in MSCs shows that red and green light reduce signal intensity, while blue light has little effect. **b** Western blot shows that red and green light increase p-AKT, p-ERK, c-MYC, KDM3A, and EZH2, and decrease H3K9me3 in MSCs. **c** Green light specifically increases p-AKT and Snail, while reducing β-catenin and H3K9me3 levels. **d** FRET analysis shows that CM from red light–treated MSCs affects intracellular signaling in MDA-MB-231 cells. **P < 0.01, ***P < 0.001, ns not statistically significant. Ctrl control, and CM conditioned medium.

Histone methylation is a critical epigenetic modification that governs gene expression by modulating chromatin architecture and accessibility. We specifically examined the role of H3K9 trimethylation (H3K9me3) in the context of iTS cell generation, focusing on its expression dynamics in response to OP stimulation. Upon irradiation with red and green pulses, conditions known to induce iTS cells, we observed a marked reduction in H3K9me3 levels (Fig. 6a, b). This decrease correlated with enhanced transcriptional activity, as evidenced by the upregulation of key signaling and epigenetic regulators, including p-Akt, p-Erk, c-Myc, KDM3A, and EZH2 (Fig. 6b, c). To further delineate the epigenetic consequences of red pulse stimulation, we utilized a FRET-based biosensor designed to detect H3K9me3. Notably, incubation of MDA-MB-231 cells with CM derived from red light-treated MSCs resulted in a significant increase in H3K9me3 levels (Fig. 6d). This finding suggests a context-dependent modulation of H3K9me3, potentially reflecting differential chromatin responses to direct OP versus paracrine signaling mediated by CM.

### Epigenetic inhibitors, TSA and Chaetocin, promoted MSC reprogramming into iTS cells by reducing H3K9me3

**Fig. 7:**
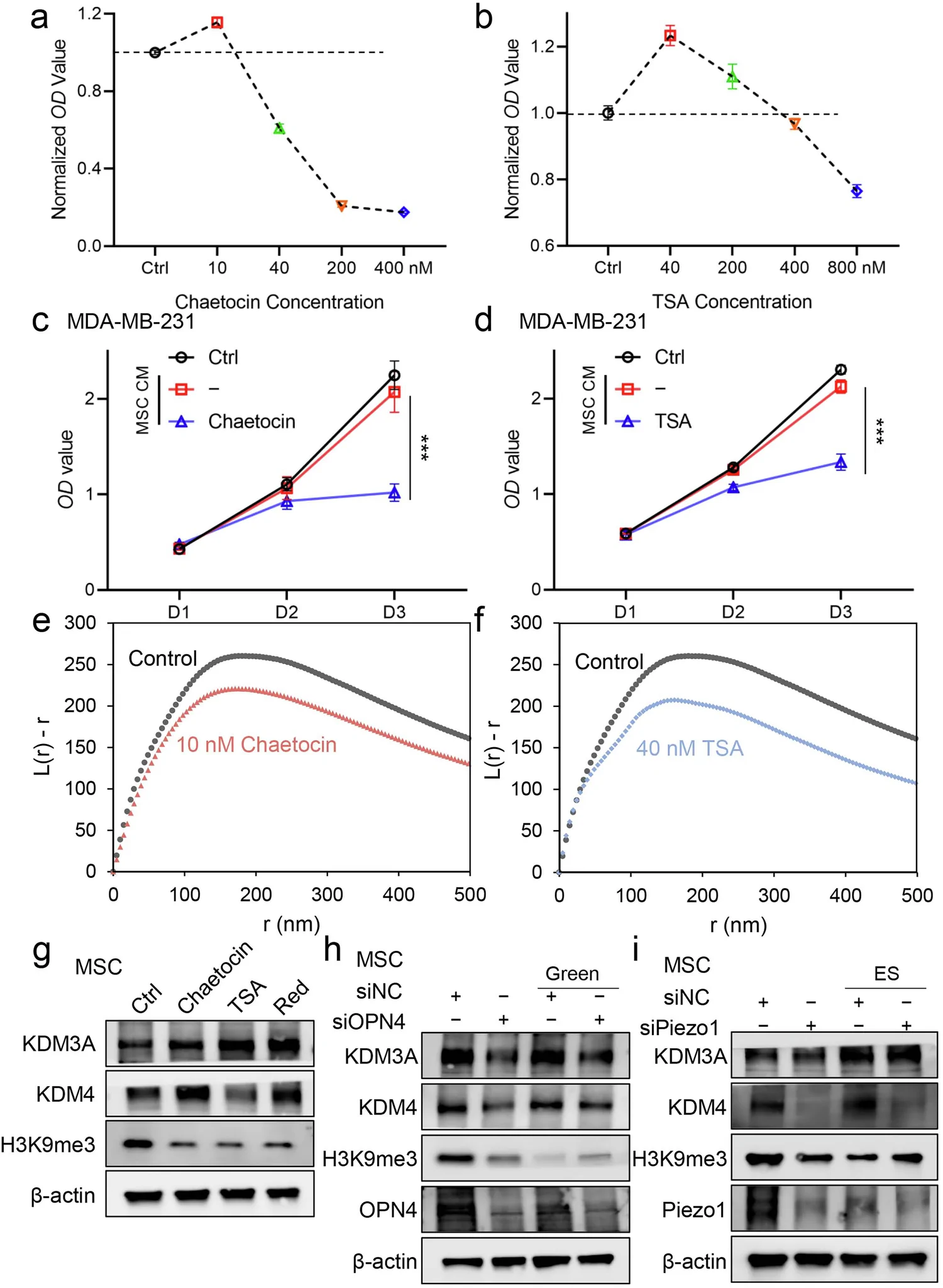
Pharmacological modulation of H3K9me3 recapitulates iTS cell induction. **a**, **b** A lower dose of Chaetocin and TSA, respectively, promoted MTT-based viability of MSCs, while higher doses are cytotoxic. **c**, **d** MSC-derived CM, treated with 10 nM Chaetocin or 40 nM TSA, respectively, suppressed the viability of MDA-MB-231 cells. **e**, **f** Reduction in nucleosome clustering in MSCs, in response to Chaetocin and TSA administration, respectively. **g** An increase in KDM3A/4 and a decrease in H3K9me3, in MSCs in response to chaetocin, TSA, and red pulses. **h**, **i** Effects of silencing OPN4 or Piezo1 in MSCs, respectively, on the levels KDM3A/4 and H3K9me3, in response to green light pulses and ES. ***P < 0.001. Ctrl control; and CM conditioned medium.

To validate the involvement of H3K9me3 in iTS cells induction, we treated MSCs with a histone methyltransferase inhibitor, Chaetocin, and a histone deacetylase inhibitor, TSA. Dose–response assays revealed that low concentrations of both agents enhanced MSC viability, whereas higher concentrations exerted dose-dependent cytotoxicity. We thus selected 10 nM Chaetocin and 40 nM TSA for the generation of iTS cells (Fig. 7a, b). CM collected from the inhibitor-treated MSCs significantly inhibited the viability of MDA-MB-231 and MDA-MB-436 cells (Fig. 7c, d and Supplementary Fig. S9). Consistent with OP-induced reprogramming, STORM imaging showed reduced nucleosome clustering (Fig. 7e, f). Moreover, these inhibitors increased KDM3A/4 and reduced H3K9me3 in MSCs, comparable to the effects of red light (Fig. 7g). Finally, the silencing of OPN4 or Piezo1 abolished these epigenetic changes under green light or ES, underscoring the requirement of sensory pathways in mediating iTS cells induction (Fig. 7h, i).

### TFRC and ANXA2 were identified as novel extracellular tumor suppressors

**Fig. 8:**
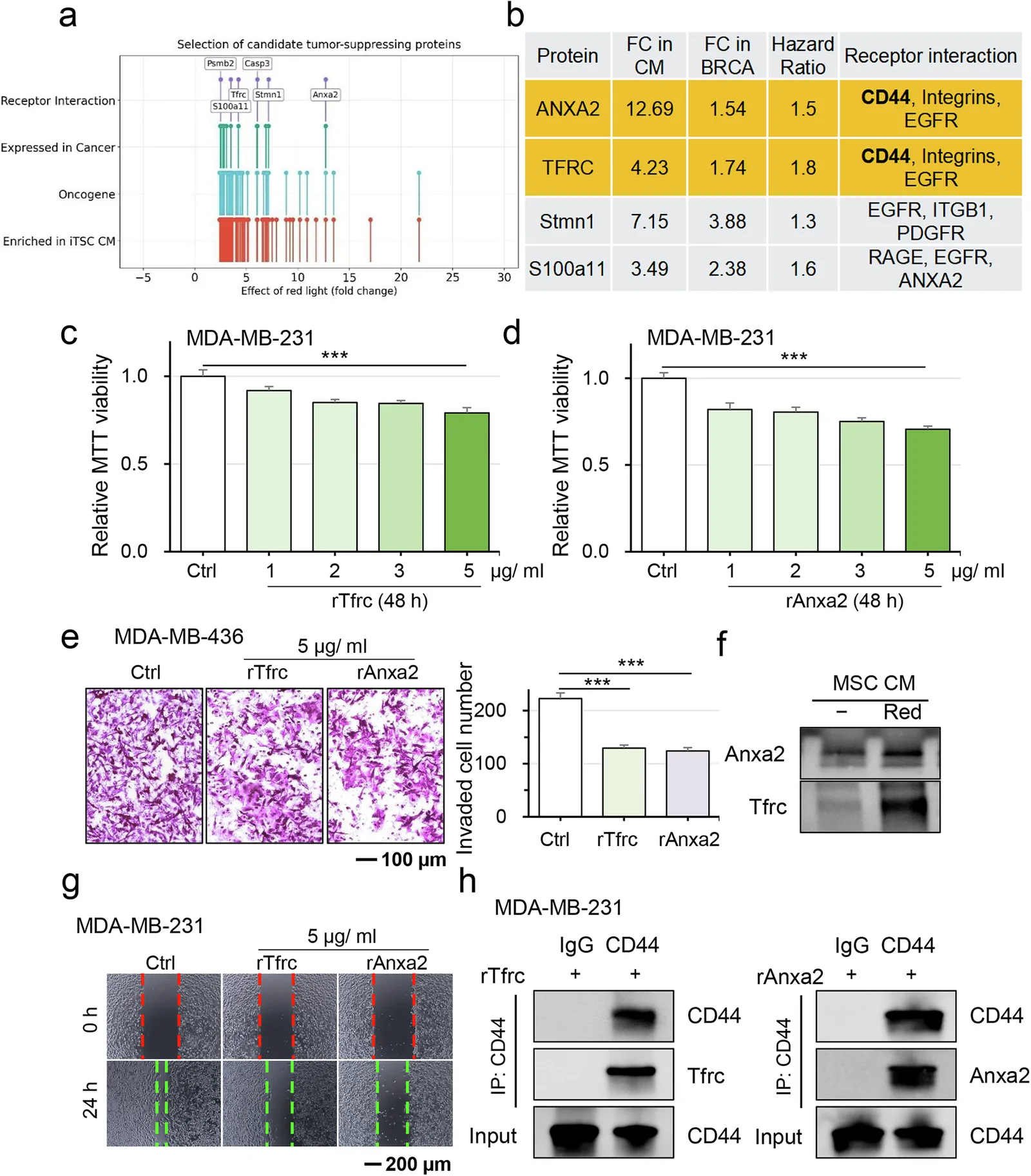
TFRC and ANXA2 acted as extracellular tumor suppressors. **a** Four selection criteria as extracellular tumor suppressors. **b** A list of candidate tumor-suppressing proteins, enriched in red-light-treated MSC-derived CM. **c**, **d** Reduction in MTT-based viability of MDA-MB-231 cells by extracellular TFRC or ANXA2, respectively. **e**, **g** Reduction in cell invasion and migration, respectively, by recombinant TFRC or ANXA2 proteins. **f** Enrichment of TFRC and ANXA2 in red-light-treated MSC-derived CM. **h** Co-immunoprecipitation of TFRC and ANXA2 with a cell surface protein, CD44. ***P < 0.001, and Ctrl control.

Previous studies on iTS cells revealed that CM derived from iTS cells were enriched with a distinct group of proteins that exhibit tumor-suppressive activity in the extracellular space, despite having oncogenic roles intracellularly. This duality, acting as extracellular suppressors and intracellular aggressors, highlights a unique regulatory paradigm in cancer biology. To identify potential extracellular tumor-suppressing proteins using mass spectrometry-based global proteomics data, we established four selection criteria: (a) Enrichment in CM derived from iTS cells; (b) established oncogenic roles within cancer cells; (c) elevated expression levels in cancer cells compared to normal cells; and (d) ability to interact with cell surface receptors. Based on these criteria, we shortlisted four candidate proteins: transferrin receptor (TFRC), annexin A2 (ANXA2), stathmin 1 (STMN1), and S100 Calcium Binding Protein A11 (S100a11) (Figs. 8a, 8b**;** Supplementary Table S1).

Functional validation using MTT assays demonstrated that TFRC and ANXA2 significantly suppressed cancer cell viability when applied extracellularly, supporting their roles as novel extracellular tumor suppressors (Figs. 8c, d). Consistently, Western blot analysis of CM from MSCs showed that red light stimulation significantly increased the secretion of ANXA2 and TFRC compared with the control (Fig. 8f). Treatment of MDA-MB-231 cells with 5 μg/ml recombinant ANXA2 or TFRC markedly suppressed cancer cell migration and invasion, as confirmed by transwell and wound-healing assays (Figs. 8e, g). In vitro co-immunoprecipitation assays confirmed that exogenous ANXA2 and TFRC interact with cancer cell–surface marker, CD44. Both proteins were detected in CD44 pull-down complexes, but not in IgG controls (Fig. 8h). These findings suggest that CD44 may serve as a docking receptor for ANXA2 and TFRC. Given its role in cell adhesion and signaling, CD44 may act as a key mediator through which ANXA2 and TFRC exert anti-tumor effects.

### Suppression of tumor progression and osteoclast development

**Fig. 9:**
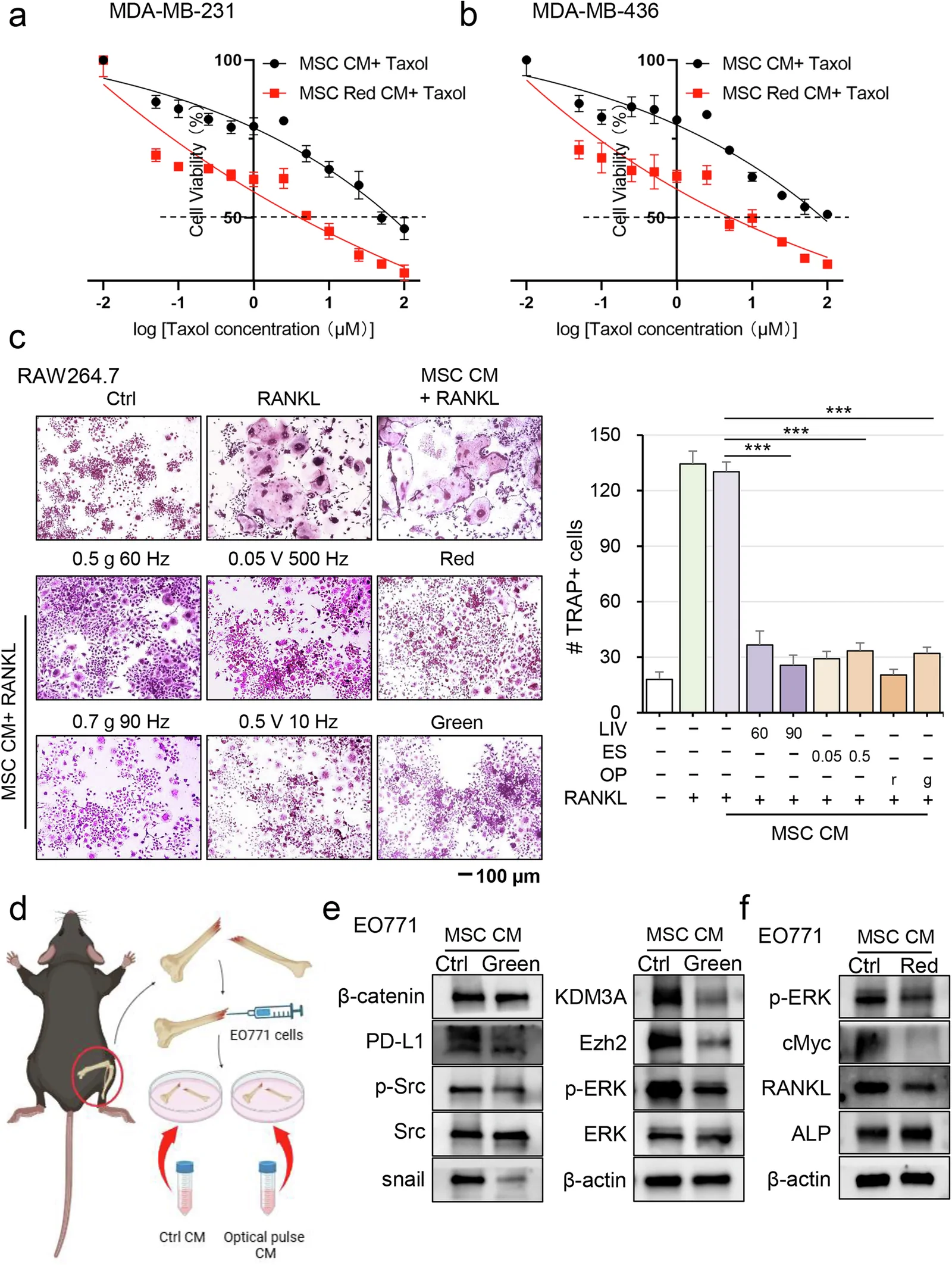
Suppression of tumor progression and osteoclast development. **a**, **b** MDA-MB-231 and MDA-MB-436 cell lines, respectively, were treated with MSC-derived CM with and without red pulse irradiation, combined with Taxol. Cell viability (MTT assay) showed that red light-treated MSC-derived CM enhanced the sensitivity of tumor cells to Taxol. **c** RAW264.7 cells were RANKL-stimulated to differentiate into osteoclasts. MSC-derived CM, stimulated by LIV, electric stimulation, or light, significantly inhibited osteoclast formation by reducing the number of TRAP-positive multinucleated cells. **d** Diagram of a murine bone metastasis model. EO771 cells were seeded on bone and treated with green/red light-treated MSC-derived CM. **e**, **f** Western blot analysis, using a mixture of bone cells and EO771 cells, isolated from the bone model. The administration of green/red light-treated MSC-derived CM reduced the level og p-Src, Snail, KDM3A, EZH2, p-ERK, PD-L1, c-Myc, and RANKL, with an increase in ALP. ***P < 0.001, Ctrl control, and CM conditioned medium.

Effective treatment of bone metastasis requires compatibility with existing therapies, alongside the dual goals of inhibiting tumor progression and preventing bone degradation mediated by osteoclasts. We demonstrated that CM derived from OP-treated MSCs can be co-administered with Taxol, significantly enhancing its antitumor efficacy (Figs. 9a, b and Supplementary Fig. S10). Additionally, this CM suppressed the maturation of RANKL-stimulated pre-osteoclasts, indicating its potential to inhibit osteoclastogenesis (Fig. 9c). Importantly, in a mouse ex vivo model of breast cancer-associated bone metastasis, the same CM markedly reduced tumor progression (Fig. 9d). Mechanistically, CM from green-treated MSCs downregulated key oncogenic and immunosuppressive markers, including β-catenin, phosphorylated Src (p-Src), Snail, phosphorylated Erk (p-Erk), PD-L1, KDM3A, and EZH2 (Fig. 9e). The reduction in PD-L1 suggests a reversal of tumor-induced immune suppression, while decreased levels of KDM3A and EZH2 point to epigenetic inhibition of oncogenic pathways. Furthermore, CM from red-treated MSCs led to decreased expression of p-Erk, c-Myc, and RANKL, accompanied by a modest increase in alkaline phosphatase (ALP), a marker associated with bone formation (Fig. 9f).

**Fig. 10:**
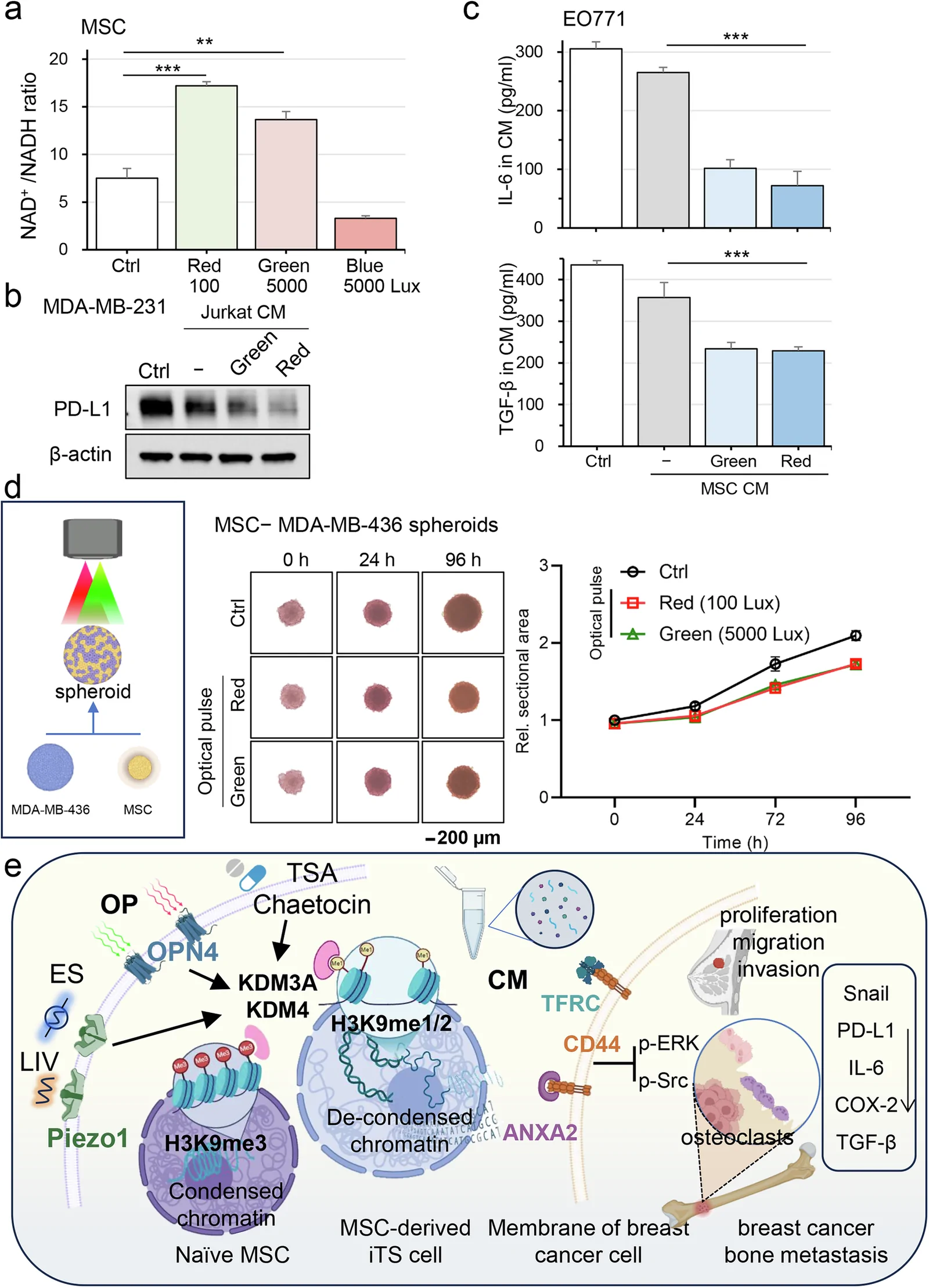
Metabolic, immune-related, and functional effects associated with optical pulses–induced iTS cell generation. **a** OP altered the metabolic state of MSCs, as shown by changes in the intracellular NAD⁺/NADH ratio. Red light markedly increased the ratio, green light induced a moderate increase, and blue light reduced it. **b**, **c** CM derived from biophysically stimulated MSCs or Jurkat T cells reduced PD-L1 expression and decreased inflammatory markers, including IL-6 and TGF-β, in tumor cells. **d** Green and red light suppressed the growth of tumor/MSC spheroids. Representative images and quantitative analysis are shown. **e** Revised working model of iTS cell generation and tumor suppression. OP act through OPN4, whereas LIV and ES act through Piezo1, to promote chromatin decondensation and transition of MSCs into iTS cells. CM derived from iTS cells contains tumor-suppressive factors, including TFRC and ANXA2, and is associated with reduced oncogenic and inflammatory signaling in tumor cells, thereby contributing to suppression of tumor progression and osteoclast-associated bone resorption. **P < 0.01, ***P < 0.001, Ctrl control, and CM conditioned medium.

To further assess whether tumor-suppressive CM also modulates immune-related signaling in tumor cells, we examined the expression of PD-L1 (programmed death-ligand 1) along with key inflammatory mediators. PD-L1 is a critical immune checkpoint ligand that engages PD-1 on immune cells, enabling tumor cells to evade immune surveillance. Western blot analysis revealed that CM derived from OP-treated MSCs or Jurkat T cells markedly reduced PD-L1 levels in breast cancer cells (Fig. 10b and Supplementary Fig. S10d), indicating that tumor-suppressive CM can attenuate immune evasive signaling within cancer cells.

Moreover, ELISA analyses showed that inflammatory markers, including IL-6 and TGF-β, were reduced in tumor cells treated with CM derived from OP-treated MSCs (Fig. 10c). These results indicate that the CM exerts multiple functions, including the suppression of inflammatory and immune-evasive signaling pathways. Together, these findings suggest that tumor-suppressive CM not only inhibits tumor cell proliferation and migration but also reshapes the immune-related phenotype of cancer cells.

Additionally, breast cancer cells (MDA-MB-436) were co-cultured with MSCs at a 4:1 ratio to form 3D tumor spheroids and treated daily with red or green light. Tumor spheroid size was monitored each day. The results showed that red or green light treatment significantly inhibited tumor growth compared to the control group. These findings further demonstrate the efficacy of OP as a non-contact therapeutic strategy and highlight the direct tumor-suppressive activity of iTS cells (Fig. 10d). Collectively, these results underscore the therapeutic potential of MSCs and MSC-derived CM in modulating both tumor and bone microenvironments. The mechanistic overview of biophysical and epigenetic modulation of iTS cell generation, as well as suppression of breast cancer progression and bone metastasis, is illustrated based on the observations in this study (Fig. 10e).

## Discussion

We have previously demonstrated multiple approaches for generating iTS cells, including the overexpression of oncogenes, such as two Yamanaka factors (c-Myc and Oct4), in combination with β-catenin, Akt, and Snail [24]. Additionally, chemical activation of proliferative signaling pathways like Wnt, PKA, and PI3K has proven effective [25]. More recently, we expanded our toolkit to include mechanical and electrical stimulations, such as low-intensity vibrations, mechanical shaking, and alternating electrical fields [4, 5, 21]. In this study, we introduce a new form of biophysical stimulation: visible light pulses. Here, we introduce visible light pulses as a novel biophysical stimulus. Successful reprogramming depended on both light wavelength and irradiation parameters, with green and red pulses effectively converting MSCs and T cells into iTS cells, whereas blue pulses did not.

As illustrated in Fig. 10e, our study reveals a biphasic mechanism by which biophysical and biochemical cues synergistically reprogram MSCs and modulate tumor-associated signaling. Biophysical stimuli, including OP, LIV, and ES, in concert with histone-modifying agents such as TSA and Chaetocin, upregulate histone demethylases KDM3A and KDM4. These enzymes catalyze the stepwise demethylation of histone H3 at lysine 9 (H3K9), thereby promoting chromatin decondensation. This epigenetic remodeling facilitates the reprogramming of MSCs into iTS cells. The CM derived from MSC-hosted iTS cells is enriched with tumor-suppressive proteins, notably TFRC and ANXA2. These factors may interact with CD44 on recipient cells and are associated with the suppression of oncogenic and osteolytic signaling pathways. Specifically, CM treatment leads to the downregulation of KDM3A, KDM4, phosphorylated ERK, phosphorylated Src, Snail, and c-Myc. This attenuation of signaling cascades contributes to both the inhibition of cancer progression and the suppression of osteoclast-mediated bone resorption.

Optimizing stimulation parameters is critical since the response is biphasic and transient: insufficient stimulation fails to induce reprogramming, whereas excessive stimulation damages cells [5, 26, 27]. Therefore, it is critical to identify conditions that enhance cellular metabolism without compromising viability. In both LIV and ES, a frequency of ~100 Hz proved effective [4, 5, 21], and in optical stimulation, pulsed light at ~10 Hz facilitated iTS cells generation, while continuous light did not.

The selection of irradiation parameters for OP warrants further investigation. OPN4 exhibits higher photosensitivity in the blue–green spectrum [28–31]. Importantly, different biophysical modalities engage distinct mechanotransduction mechanisms. LIV and ES typically require higher frequencies (~100 Hz) [32, 33] to generate sufficient activation of mechanosensitive ion channels and to induce cytoskeletal oscillations [34]. In contrast, OP relies on receptor-mediated phototransduction [35], for which lower pulse frequencies (e.g., 10 Hz) may be adequate to trigger downstream signaling. These observations highlight the modality-specific nature of biophysical signal transduction [36]. A more systematic analysis may help identify the most effective wavelength-dependent frequency parameters for OP.

CM from green- or red-light-treated cells exhibited tumor-suppressive activity and inhibited osteoclast maturation. Combining CM with Taxol enhanced suppression of tumor progression, supporting the combinatorial utility of light-treated iTS cell-derived CM for bone-metastatic breast cancer.

Cellular plasticity allows reprogramming via biophysical stimuli, including LIV, ES, and OP. This process involves transient chromatin changes, particularly nucleosome scattering [14, 37]. In MSC-derived iTS cells, chromatin appeared more relaxed after stimulation, which may enhance chromatin accessibility and transcription. Future studies are needed to examine if similar chromatin changes occur in iTS cells generated by other methods, such as oncogene overexpression or activation of proliferative signaling such as PI3K. Regarding tumor-suppressing proteins in iTS cell-derived CM, TFRC and ANXA2 were identified to interact with CD44, a pivotal receptor in cancer progression [38–42]. Intracellularly, TFRC promotes iron uptake [43], and ANXA2 supports cytoskeletal remodeling and angiogenesis [44, 45], while CD44 regulates cell stemness and EMT [46, 47].

In contrast to their intracellular roles, extracellular TFRC and ANXA2 exhibited tumor-suppressive effects, highlighting their context-dependent functions. Previous studies have demonstrated that stromal and microenvironmental cues can modulate the proliferation and survival of cancer cells [48, 49]. Consistent with these observations, our previous studies demonstrated that iTS cell–derived CM from diverse cellular sources exhibit robust tumor-suppressive activities [50, 51]. Although we demonstrated extracellular interaction between TFRC/ANXA2 and CD44, the precise downstream signaling cascades triggered by this interaction remain to be fully characterized.

Extreme chromatin decondensation may foster oncogenic transformation, while moderate decondensation may facilitate cellular reprogramming toward a tumor-suppressive phenotype [52, 53]. This study demonstrated that histones are central to the regulatory balance. The repressive mark H3K9me3, emphasized in this study, contributes to heterochromatin compaction and silencing of tumor suppressor genes. In contrast, acetylation marks such as H3K9ac promote chromatin relaxation, enhancing oncogene activation. Aggressive breast cancer subtypes often exhibit widespread chromatin loosening marked by diminished repressive histone marks and enhancer reprogramming [54, 55]. While this study focused on the regulation of H3K9 through two histone-modifying enzymes, KDM3A and KDM4, numerous other epigenetic marks and regulatory enzymes, including histone deacetylases, may also contribute to the generation of iTS cells and the progression of breast cancer-associated bone metastasis. Further comprehensive analyses are warranted to elucidate the broader landscape of chromatin modifications involved in these processes.

OPs present a minimally invasive approach for reprogramming MSCs and T cells into iTS cells. To improve tissue penetration and reduce thermal effects, infrared light may offer a more suitable alternative to conventional visible wavelength sources [56, 57]. Enhancing the expression of photosensitive receptors such as OPN4 could further increase the efficiency of iTS cell induction. Translational advancement of this strategy will require rigorous preclinical validation. To deliver OP to in situ tumorigenic regions, including bone metastatic sites, the introduction of optical fibers may be necessary. Additionally, this approach could be extended to other cancer types, such as skin and esophageal cancers, where direct light exposure is more readily achievable.

Together, these findings underscore the translational promise of OP-mediated iTS cell generation as a dual-action therapeutic strategy. By modulating light parameters and leveraging OPN4-driven reprogramming, this approach not only reshapes the tumor microenvironment but also confers bone-protective effects. While further validation is warranted, the capacity to target both primary breast tumors and their skeletal metastases marks a meaningful step toward more integrated and minimally invasive treatment paradigms for metastatic breast cancer.

## Supplementary information

Supplementary Information
