# Supplementary material for: Visible light reprograms MSCs and T cells into tumor-suppressive states via OPN4-mediated epigenetic remodeling
Source: Acta Pharmacol Sin. 2026 Jul 6;47(10):3042–58. doi: 10.1038/s41401-026-01851-4 (PMC13586407; doi:10.1038/s41401-026-01851-4)
Supplement: Supplementary file 1 [file 41401_2026_1851_MOESM1_ESM.docx]

Supplementary information

**Supplementary Table S1** Selection of tumor-suppressing proteins.

| Protein | Fold change in conditioned medium^1^ | Fold change in breast cancer^2^ | Hazard Ratio for overall survival^3^ | | Candidate interacting receptors |
| --- | --- | --- | --- | --- | --- |
| ANXA2 | 12.69 | 1.54 | 1.50 | CD44, Integrins, EGFR | |
| TFRC | 4.23 | 1.74 | 1.80 | CD44, Integrins, EGFR | |
| Stmn1 | 7.15 | 3.88 | 1.30 | EGFR, ITGB1, PDGFR | |
| S100a11 | 3.49 | 2.38 | 1.60 | RAGE, EGFR, ANXA2 | |
| Psmb2 | 2.50 | 1.76 | 1.90 | TNFR1, EGFR | |
| Casp3 | 6.07 | 1.89 | 1.40 | FAS, TNFR1, DR4/DR5 | |
| Tuba1c | 3.06 | 3.48 | 1.90 | Integrins, EGFR, TFRC | |
| Nutf2 | 2.84 | 1.52 | 1.70 | NUPs | |
| GADPH | 2.46 | 2.20 | 1.70 | EGFR | |
| Mcm5 | 2.73 | 1.53 | 1.50 | EGFR, AR | |
| Cct6a | 6.89 | 1.64 | 1.40 | EGFR, IGF1R, GPCRs | |
| Psmd12 | 6.02 | 1.72 | 1.40 | EGFR, TNFR1, TCR | |
| Ppm1g | 2.55 | 1.74 | 1.40 | GR | |
| Dsp | 21.73 | 3.57 | 0.94 | DEG1-4, DSC1-3 | |
| Jup | 13.46 | 1.99 | 1.20 | DEG1-4/DSC1-3 | |
| CTSD | 10.88 | 2.35 | 1.10 | M6PR, LRP1 | |
| Psma4 | 10.18 | 1.66 | 1.10 | TNFR1, EGFR | |
| TXN | 8.85 | 2.00 | 1.20 | IL-1R, TLR4, TFRC, EGFR | |
| Sec23b | 7.03 | 2.54 | 0.78 | EGFR, INSR, TFRC | |
| Snrpd3 | 6.66 | 2.04 | 0.93 | IL7R, EGFR, FGFR2 | |
| Txndc5 | 5.11 | 1.59 | 1.20 | PERK, ATF6, Integrins | |
| HNRNPF | 4.63 | 2.03 | 0.97 | FGFR2, CD44 TCR | |
| Eif2s1 | 4.54 | 1.52 | 1.20 | PERK, IFNAR1/2, TNFR1 | |
| Cdh2 | 4.41 | 1.69 | 1.10 | Cadherins, EGFR, Intrgrins | |
| Ugdh | 4.10 | 2.46 | 1.00 | CD44, TLR4, RHAMM | |
| Copb2 | 3.98 | 2.29 | 1.10 | EGFR, INSR, TFRC | |
| Dhx9 | 3.69 | 1.63 | 0.94 | TLR9 | |
| Ppt1 | 3.60 | 2.52 | 0.99 | EGFR, TLR4, CD44 | |
| Adss2 | 3.39 | 1.80 | 0.98 | P2RX7 | |
| Copz1 | 3.36 | 2.26 | 1.00 | EGFR, INSR, TFRC | |
| Gsr | 3.25 | 2.31 | 0.96 | EGFR, TLR4, TGFBR1/2 | |
| Trim28 | 3.12 | 1.81 | 1.10 | ERα | |
| Rps10 | 2.81 | 1.66 | 1.10 | TLR4, EGFR, TNFR1, ERα | |
| Psma2 | 2.71 | 1.65 | 0.88 | EGFR, TNFR1, TCR | |
| Eif4e | 2.71 | 1.79 | 1.10 | EGFR, INSR | |
| Hspa1b | 2.62 | 2.14 | 1.10 | TLR2, CD91 | |
| Ahcy | 2.60 | 3.27 | 1.10 | ERα, GR | |
| Mtpn | 2.46 | 1.62 | 1.10 | NF-kB | |
| Arpc4 | 2.42 | 1.65 | 1.00 | ITGB1, EGFR | |
| Hspd1 | 2.40 | 1.70 | 1.20 | TLR2/4, CD14 | |
| H2ac4 | 17.02 | — | — | — | |
| Nccrp1 | 11.74 | — | 1.20 | NK cell receptors | |
| Actc1 | 9.50 | 0.57 | 1.10 | Integrins, DAG1 | |
| Ddx39b | 9.50 | 0.91 | 0.89 | IL7R | |
| GSTP1 | 9.22 | 0.53 | 1.50 | TLR4, EGFR, TNFR1 | |
| Apoa1 | 7.92 | 0.85 | 1.20 | SR-B1, ABCA1, ABCG1 | |
| Ahnak | 7.50 | 0.52 | 1.00 | CACNA1C, TGFBR1, EGFR | |
| Hcfc1 | 6.84 | 1.42 | 1.20 | EGFR, IGF1R, GFGFR1 | |
| Pkp1 | 6.53 | 0.22 | 0.94 | DEG1-4, DSC1-3 | |
| Rpl26 | 5.18 | 0.74 | 0.69 | FAS, TNFR1, DR5, EGFR, IGF1R | |
| Idh1 | 5.09 | — | 1.30 | EGFR, PDGFRA, INSR | |
| Rps3 | 4.84 | 0.82 | 1.00 | TNFR1, TLR4, EGFR | |
| Epdr1 | 4.75 | 0.48 | 0.84 | Integrins | |
| Anxa1 | 4.39 | 0.22 | 1.40 | FPR1, EGFR | |
| Eif4g1 | 4.10 | 1.36 | 1.20 | EGFR, INSR | |
| Srsf3 | 4.03 | — | 1.10 | FGFR2, CD44, INSR | |
| Pdcd6ip | 4.00 | 0.64 | 1.20 | EGFR, FAS | |
| Psmd5 | 4.00 | 1.44 | 1.20 | EGFR, THFR1, TCR | |
| Ptbp1 | 3.97 | 1.29 | 1.20 | — | |
| Cbr1 | 3.64 | — | 1.30 | AHR, ERα | |
| Rpl7a | 3.59 | 1.16 | 0.68 | THR, Erα | |
| Otub1 | 3.53 | 1.37 | 1.40 | EGFR, TGFRB1, TNFR1 | |
| Prdx5 | 3.51 | 1.33 | 1.00 | TLR4/TLR2 | |
| Rplp0 | 3.39 | 1.16 | 1.10 | EGFR, IGF1R | |
| Apoh | 3.37 | — | 1.40 | LRP8, TLR2/TLR4, ANXA2 | |
| Rab11b | 3.35 | 0.73 | 0.96 | TFRC, EGFR, ADRB2 | |
| Samhd1 | 3.34 | 0.68 | 1.20 | INFAR1/2, CD4 | |
| Creg1 | 3.34 | 1.01 | 1.10 | M6PR | |
| Naglu | 3.32 | 0.74 | 0.80 | M6PR, HSPG receptors | |
| Fabp5 | 3.27 | 0.29 | 1.60 | PPARβ/δ, RARs | |
| Psmb1 | 3.21 | 1.23 | 0.97 | TNFR1, EGFR, TCR | |
| Hnrnph1 | 3.17 | 0.68 | 0.94 | FRFR2, CD44, INSR | |
| Rps13 | 3.13 | 0.93 | 0.75 | EGFR, TLR4, ERα | |
| Pdcd5 | 3.04 | 1.22 | 1.60 | CD95, TNFR1 | |
| Wars1 | 3.03 | 1.20 | 1.10 | KDR, TLR2 | |
| Arpc2 | 3.00 | 1.20 | 1.70 | ITGB1, EGFR, TCR | |
| Psmd3 | 3.00 | 1.40 | 1.30 | EGFR, TNFR1, TCR | |
| Tes | 2.96 | 0.27 | 1.10 | ITGB1, EGFR, PDGFR | |
| Pcmt1 | 2.96 | 1.39 | 0.83 | EGFR, INSR | |
| Cfl2 | 2.82 | 0.34 | 1.10 | Integrins, EGFR, INSR | |
| Tpt1 | 2.81 | 0.86 | 0.80 | HRH4, TLR4 CD47 | |
| Ybx1 | 2.80 | 1.41 | 1.90 | EGFR, ERα, TLR4 | |
| Asns | 2.79 | 1.14 | 1.30 | INSR, EGFR | |
| Acp1 | 2.74 | 1.50 | 1.10 | INSR, PDGFR, EGFR | |
| Ewsr1 | 2.74 | — | 0.97 | IGF1R, PDGFR | |
| Strap | 2.73 | 1.41 | 1.00 | TGFBR1/2 | |
| Adprh | 2.70 | 0.67 | 0.90 | EGFR, TLR4 | |
| Etf1 | 2.69 | — | 1.10 | EGFR, INSR | |
| Hexa | 2.69 | — | 0.86 | M6PR, GM2 | |
| Nasp | 2.68 | 0.97 | 1.30 | ERα, AR | |
| Ipo5 | 2.64 | 0.93 | 1.10 | NUPs | |
| Psma1 | 2.63 | 1.24 | 1.10 | TNFR1, EGFR, tcr | |
| Thbs4 | 2.62 | — | 0.89 | Inregrins, CD47, LRP1 | |
| Septin2 | 2.57 | — | 1.00 | EGFR, ITGB1, PDGFR | |
| Nedd8 | 2.56 | 1.33 | 0.96 | EGFR, TGFBR1/2, TNFR1 | |
| Septin11 | 2.53 | 0.77 | 1.20 | EGFR, ITGB1, PDGFR | |
| Eif3l | 2.50 | 0.76 | 0.59 | EGFR, INSR | |
| Xpo1 | 2.48 | 1.38 | 1.30 | NUPs | |
| Serpinb1a | 2.43 | — | — | TLR4, CXCR1/2 | |
| H2-K1 | 2.41 | — | — | TCR | |

^1^The optical pulse-driven fold-change of the proteins in the conditioned medium.

^2^The fold-change of the transcripts in breast cancer tissues, compared to normal tissues in the TCGA data. The sign, “—,” represents the case in which data are included in the TCGA.

^3^The hazard ratio is the ratio of overall death between the high expression group (50% or more than the average) and the low expression group (50% or less than the average) of breast cancer patients in the TCGA data. The sign, “—,” represents the case in which data are included in the TCGA.

**Supplementary Figure S1. Low-intensity vibrations (LIV) reprogram MSCs into induced tumor-suppressing (iTS) cells.** LIV was applied at frequencies of 30, 60, or 90 Hz with peak accelerations ranging from 0.3× to 0.9× Earth’s gravity (*g*). (a) LIV treatment did not significantly affect the MTT-based viability of MSCs after 48 h. (b) CM from LIV-treated MSCs significantly reduced the MTT-based viability of MDA-MB-231 breast cancer cells after 48 h. (c) LIV-treated MSC-derived CM inhibited the 2D motility of MDA-MB-436 breast cancer cells within 24 h. (d) LIV-treated MSC-derived CM significantly suppressed the transwell invasion of EO771 mammary tumor cells after 24 h. (e) LIV-treated MSC-derived CM reduced the size of tumor spheroids over 96 h. ****P* < 0.001. Ctrl = control; and CM = conditioned medium.

**Supplementary Figure S2. Electrical stimulation (ES) reprograms MSCs into induced tumor-suppressive (iTS) cells.** MSCs were exposed to ES at 500 Hz (0.05 V), 100 Hz (0.1 V), or 10 Hz (0.5 V). Conditioned medium was collected after 48 h. (a) ES at 0.5 V across frequencies of 10, 100, and 1000 Hz induced a frequency-dependent reduction in MTT-based viability of MDA-MB-436 breast cancer cells after 48 h. (b) CM from ES-treated MSCs significantly decreased the viability of MDA-MB-436 cells at 48 h. (c) ES-treated MSC-derived CM inhibited the 2D motility of MDA-MB-436 cells within 24 h. (d) ES-treated MSC-derived CM significantly suppressed the invasive capacity of MDA-MB-231 cells in a transwell invasion assay after 24 h. (e) ES-treated MSC-derived CM markedly reduced tumor spheroid formation by MDA-MB-436 cells over 96 h. ***P* < 0.01, ****P* < 0.001. Ctrl = control; and CM = conditioned medium.

**Supplementary Figure S3. Tumor-suppressive capability of LIV-treated MSC-derived CM.** (a and b) Reduction of MTT-based cell viability of MDA-MB-231 breast cancer cells and 4T1.2 mammary tumor cells in response to LIV-treated MSC-derived CM. (c) Decrease in 2-dimensional motility of MDA-MB-231 cells in response to LIV-treated MSC-derived CM. ****P* < 0.001. Ctrl = control; CM = conditioned medium.

**Supplementary Figure S4. Tumor-suppressive capability of ES-treated MSC-derived CM.** (a) Frequency-dependent response of MTT-based viability of Jurkat T cells to ES with 0.01 V at 1K Hz to 100K Hz. (b) Reduction in MTT-based viability of MDA-MB-231 breast cancer cells in response to ES-treated Jurkat T-cell-derived CM at 0.01 to 0.5 V at 10 to 1000 Hz. (c) Frequency-dependent response of MTT-based viability of MSCs to ES with 0.01 V at 1K Hz to 100K Hz. (d) Reduction in MTT-based viability of MDA-MB-231 breast cancer cells in response to ES-treated MSC-derived CM at 0.01 to 0.5 V at 10 to 1000 Hz. (e) Decrease in 2-dimensional motility of MDA-MB-231 cells in response to ES-treated MSC-derived CM (0.01 V at 1000 Hz and 0.5 V at 10 Hz). ****P* < 0.001. ES = electrical stimulation; Ctrl = control; and CM = conditioned medium.

**Supplementary Figure S5. Color-dependent alterations in MTT-based viability in response to optical pulses (OP) of T cells and breast cancer cells.** The OP conditions were 1000 to 10,000 Lux at 10 Hz. (a) Responses of MDA-MB-231 breast cancer cells. (b) Responses of MDA-MB-436 cells. MTT-based viability of EO771 cells (c) and MSCs (d) after 10 Hz red, green, or blue light stimulation. (e) Responses of Jurkat T cells. (f) Responses of EL4 T cells.

**Supplementary Figure S6. Generation of iTS cells by the irradiation of red and green pulses to MSCs.** (a and b) Reduction in MTT-based viability of 4T1.2 and EO771 mammary tumor cells, respectively, by red and green OP-treated MSC-derived CM. The effect of red and green is stronger than that of blue. (c) Decrease in scratch-based motility of MDA-MB-231 breast cancer cells by red and green OP-treated MSC-derived CM. (d) Reduction in MTT-based viability of 4T1.2 mammary tumor cells by red and green OP-treated Jurkat T-cell-derived CM. (e) Reduction in MTT-based viability of EO771 mammary tumor cells by red and green OP-treated Jurkat T-cell-derived CM. **P* < 0.05, ***P* <0.01, ****P* < 0.001, ns = not statistically significant. Ctrl = control; and CM = conditioned medium.

**Supplementary Figure S7. Combined tumor-suppressive effects in response to simultaneous application of OP and LIV.** OP was given by red pulses with 100 Lux, while LIV was applied with 0.7× *g* (acceleration equivalent to Earth’s gravity) at 90 Hz. (a–d) Reduction in MTT-based viability of MDA-MB-231, MDA-MB-436, 4T1.2, and EO770 cells, respectively, by MSC-derived CM, treated by OP, LIV, and OP+LIV. (e–h) Reduction in MTT-based viability of MDA-MB-231, MDA-MB-436, 4T1.2, and EO770 cells, respectively, by T cell-derived CM, treated by OP, LIV, and OP+LIV. **P* < 0.05, ***P* <0.01, ****P* < 0.001. Ctrl = control; CM = conditioned medium.

**Supplementary Figure S8. Piezo1 and OPN4 mediate stimulus-specific reprogramming of MSCs and T cells.** (a and b) CM, derived from ES/LIV-treated MSCs, suppressed the MTT-based viability of MDA-MB-231 and MDA-MB-436 cells, whereas Piezo1 silencing abolishes this effect. (c) CM, derived from ES-treated MSCs, inhibited scratch-based migration of 4T1.2 cells, but CM, derived from Piezo1-deficient MSCs, did not. (d) OPN4-silenced Jurkat T cell-derived CM, in response to red or green pulses, lost tumor-suppressive capability without reducing MTT-based viability of two breast cancer cell lines. (e) Piezo1 silencing did not impair the tumor-suppressive capability of red/green light-treated MSC-derived CM, consistent with the stimulus-specific roles of Piezo1 and OPN4. ****P* < 0.001, ns = no statistical significance. Ctrl = control; and CM = conditioned medium.

**Supplementary Figure S9. Chaetocin and TSA converted MSCs into iTS cells, and their CM reduced the viability of MDA-MB-436 breast cancer cells.** CM was collected from MSCs treated with 10 nM Chaetocin or 40 nM TSA. (a) Reduction of viability by Chaetocin-treated MSC-derived CM. (b) Reduction of viability by TSA-treated MSC-derived CM. ****P* < 0.001, Ctrl = control; and CM = conditioned medium.

**Supplementary Figure S10. Additional analyses of metabolic activation and tumor-suppressive effects associated with optical stimulation and conditioned medium (CM).** (a) Combined effects of MSC-derived CM and paclitaxel (Taxol) on 4T1.2 mammary tumor cells. Taxol was applied over a logarithmic concentration range (−2 [0.01 μM] to +2 [100 μM]). (b) Changes in the intracellular NAD⁺/NADH ratio in MSCs following OP with or without OPN4 silencing. Red light increased the ratio in control cells, whereas this effect was attenuated in siOPN4-treated cells. (c) JC-1 fluorescence staining showing mitochondrial membrane potential in MSCs after OP with or without OPN4 silencing. Red light increased mitochondrial activity, and this effect was reduced by OPN4 knockdown. Scale bar = 50 μm. (d) Western blot analysis of PD-L1 expression in tumor cells treated with CM derived from optically stimulated MSCs. CM treatment reduced PD-L1 expression. (e) ELISA analysis of COX-2 levels in tumor cells treated with MSC-derived CM. CM treatment decreased COX-2 expression. ****P* < 0.001, ns = no statistical significance. Ctrl = control; CM = conditioned medium; OP = optical pulses.
